# Replacing Nitrogen by Sulfur: From Structurally Disordered Eumelanins to Regioregular Thiomelanin Polymers

**DOI:** 10.3390/ijms18102169

**Published:** 2017-10-17

**Authors:** Mariagrazia Iacomino, Juan Mancebo-Aracil, Mireia Guardingo, Raquel Martín, Gerardino D’Errico, Marco Perfetti, Paola Manini, Orlando Crescenzi, Félix Busqué, Alessandra Napolitano, Marco d’Ischia, Josep Sedó, Daniel Ruiz-Molina

**Affiliations:** 1 Department of Chemical Sciences, University of Naples “Federico II”, Via Cintia 4, 80126 Naples, Italy; mariagrazia.iacomino@unina.it (M.I.); gerardino.derrico@unina.it (G.D.); marco.perfetti@unina.it (M.P.); pmanini@unina.it (P.M.); orlando.crescenzi@unina.it (O.C.); alesnapo@unina.it (A.N.); 2 Catalan Institute of Nanoscience and Nanotechnology (ICN2), CSIC and The Barcelona Institute of Science and Technology, Campus UAB, 08193 Bellaterra, Spain; juan.mancebo@icn2.cat (J.M.-A.); mireia.guardingo@gmail.com (M.G.); josep.sedo@icn2.cat (J.S.); 3 Department de Química, Universitat Autònoma de Barcelona (UAB), Campus UAB, 08193 Bellaterra, Spain; rkl.martin@gmail.com (R.M.); Felix.Busque@uab.cat (F.B.)

**Keywords:** eumelanin, polydopamine, bioinspired polymers, bioinspired coatings, thiomelanin

## Abstract

The oxidative polymerization of 5,6-dihydroxybenzothiophene (DHBT), the sulfur analog of the key eumelanin building block 5,6-dihydroxyindole (DHI), was investigated to probe the role of nitrogen in eumelanin build-up and properties. Unlike DHI, which gives a typical black insoluble eumelanin polymer on oxidation, DHBT is converted to a grayish amorphous solid (referred to as thiomelanin) with visible absorption and electron paramagnetic resonance properties different from those of DHI melanin. Mass spectrometry experiments revealed gradational mixtures of oligomers up to the decamer level. Quite unexpectedly, nuclear magnetic resonance (NMR) analysis of the early oligomer fractions indicated linear, 4-, and 7-linked structures in marked contrast with DHI, which gives highly complex mixtures of partially degraded oligomers. Density functional theory (DFT) calculations supported the tendency of DHBT to couple via the 4- and 7-positions. These results uncover the role of nitrogen as a major determinant of the structural diversity generated by the polymerization of DHI, and point to replacement by sulfur as a viable entry to regioregular eumelanin-type materials for potential applications for surface functionalization by dip coating.

## 1. Introduction

Soft functional polymers mimicking the black eumelanin pigments of human skin, hair, and eyes, as well as cephalopod ink, are the focus of growing interest in materials science, bioengineering, and biomedicine because of their biocompatibility and bioavailability combined with broadband visible light absorption, hydration-dependent semiconductor properties, and antioxidant and free radical behavior [1,2,3,4,5,6,7], which suggest applications in organic electronics, bioelectronics, light-harvesting, and coating [8,9,10,11,12,13]. Traditionally, black insoluble eumelanin-like materials have been obtained by chemical or enzymatic polymerization of 3,4-dihydroxyphenylalanine (DOPA), 5,6-dihydroxyindole (DHI) or 5,6-dihydroxyindole-2-carboxylic acid (DHICA) [14,15].

Since 2007, polydopamine (PDA) has occupied a relevant place in materials science. It is a mussel byssus-inspired eumelanin-type polymer that displays unusual underwater adhesion properties and is widely used for surface functionalization and coating [8]. Material-independent adhesion layers produced with PDA can be exploited for the immobilization of biological molecules, the production of self-assembled monolayers, nanoparticle modification, and fouling-resistant systems [16,17,18,19,20,21]. The processes and mechanisms underlying the deposition of PDA coatings have been investigated in detail [16,22,23], and the importance of specific cooperative effects between functional groups in the adhesion mechanisms has been emphasized, including mainly the coexistence of catechol/quinone and primary amine functionalities [24,25,26,27].

**Figure 1:**
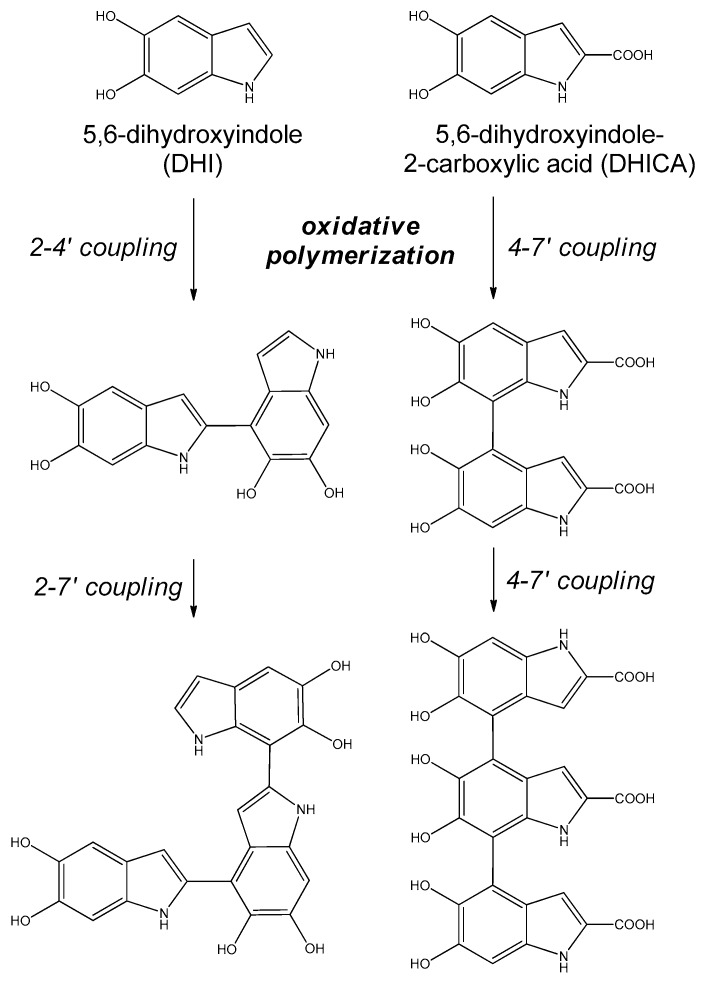
Representative structures of the main oligomers isolated by the oxidation of 5,6-dihydroxyindole (DHI) and 5,6-dihydroxyindole-2-carboxylic acid (DHICA), highlighting basic differences in modes of coupling and oligomer growth.

**Figure 2:**
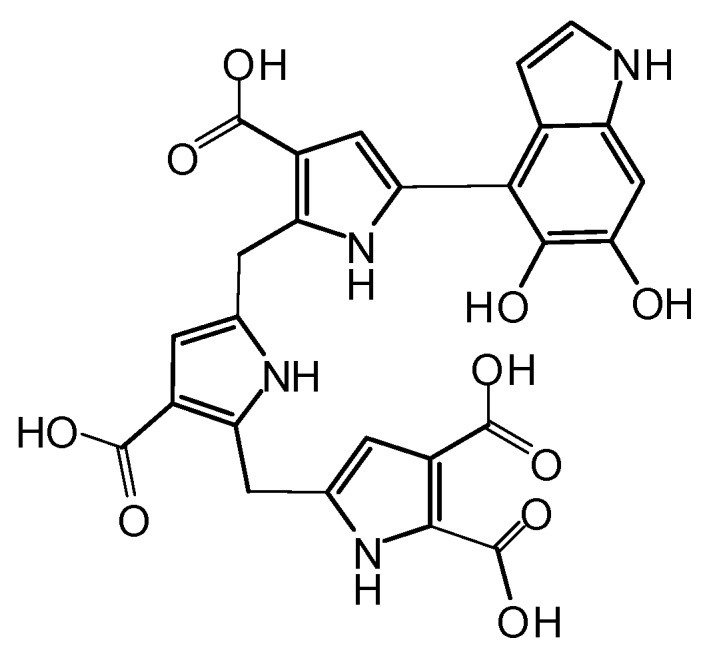
Structure exemplifying oxidative breakdown of indole units in representative DHI-based eumelanin components leading to pyrrolecarboxylic acid moieties.

However, despite rapid advances, implementing versatile and competitive technological solutions based on eumelanins is currently a considerable challenge. To a considerable degree, progress toward a definitive maturation of the field is hindered by the high insolubility and amorphous character of these materials, and their unusual structural complexity and heterogeneity arising by manifold levels of chemical disorder [28]. The main consequence is that eumelanin physicochemical properties cannot be framed within well-defined sets of structure–property–function relationships, which makes it difficult to develop eumelanin-based functional materials with tailored properties by rational chemical manipulation. Extensive structural investigations have demonstrated that eumelanin heterogeneity stems in part from the irregular mode of polymerization of DHI via mainly 2,4′- and 2,7′-bondings, but also 2,3′-, 3,3′- and 4,4′-bondings at the dimer coupling level, which gives rise to a variety of structural scaffolds characterizing each oligomer population (Figure 1). Furthermore, mass spectrometric studies underscored the marked tendency of DHI oligomers to undergo oxidative breakdown with the formation of pyrrolecarboxylic acid moieties (Figure 2).

At variance with DHI, DHICA, the main building block in natural melanins, polymerizes mainly via the 4- and 7-positions, leading to linear oligomeric scaffolds due to the deactivating effect of the carboxyl group on the pyrrole moiety. As a result, DHICA melanin shows lower visible light absorption, but much stronger antioxidant properties compared with DHI melanin. Controlling chemical disorder and orienting DHI polymerization toward well-defined structures that exhibit specific properties without making recourse to functional groups is therefore a major goal in eumelanin research.

An issue of considerable scientific and practical relevance that has remained so far unexplored relates to the actual role of nitrogen in determining the mechanisms of build-up and the main physicochemical properties of eumelanin-type materials. In particular, in order to expand the current framework of structure–property relationships, it was important to assess to what extent nitrogen is responsible for the specific oxidation behavior and mode of coupling of DHI and dopamine.

**Figure 3:**
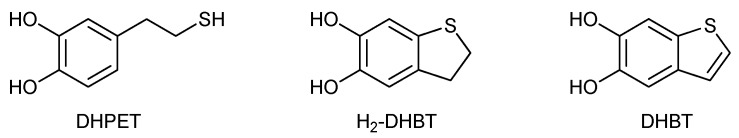
Structures of the three sulfur analogs of eumelanin building blocks investigated in this study.

In an attempt to address this central issue in eumelanin-based materials, we have undertaken herein a systematic investigation of the synthesis and oxidative polymerization of 2-(3,4-dihydroxyphenyl)ethanethiol (DHPET), 2,3-dihydro-5,6-dihydroxybenzo[b]thiophene (H_2_-DHBT) and 5,6-dihydroxybenzo[b]thiophene (DHBT), the sulfur-containing analogs of three eumelanin building blocks, namely dopamine, leucodopaminochrome, and DHI (Figure 3).

Besides the spectral, morphological, and chemical characterization of the polymeric material produced by the oxidation of DHBT, for which the term thiomelanin is proposed, the study reports a detailed quantum chemical characterization of H_2_-DHBT, DHBT, and their oxidation products, in order to assess the potential of nitrogen replacement by sulfur for eumelanin manipulation toward novel eumelanin-type materials.

## 2. Results and Discussion

### 2.1. Synthesis and Oxidation of DHPET (***1***)

In preliminary experiments, 2-(3,4-dihydroxyphenyl)ethanethiol, the thia-analog of dopamine, DHPET, was synthesized, and its oxidation chemistry was investigated under different conditions. Besides drawing a more defined set of structure–property–function relationships in eumelanin-type materials, the specific aims of the experiments were:

- to verify the feasibility of this approach as an expedient entry to DHBT via nucleophilic attack of the thiol (-SH) group onto the *o*-quinone groups;
- to assess whether a PDA-like material is formed regardless of DHBT formation, and to investigate its absorption, free radical, and adhesion properties.

**Figure 4:**
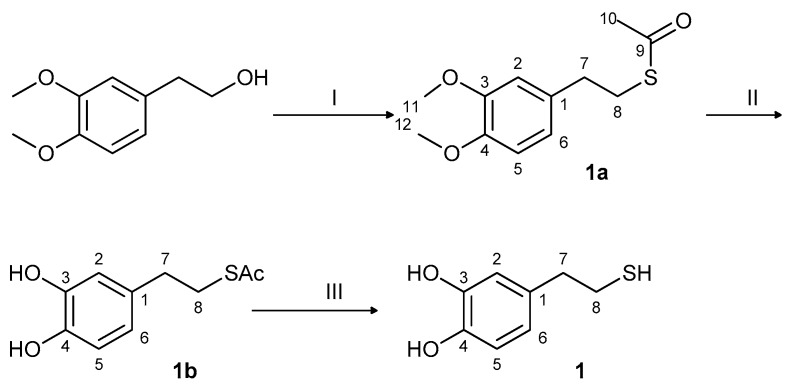
2-(3,4-dihydroxyphenyl)ethanethiol (DHPET, (**1**) synthetic route. **I**: 1) MsCl, Et_3_N/ Tetrahydrofuran (THF) dry, N_2_ 2) CH_3_COSK/ Dimethylformamide (DMF); **II**: BBr_3_/ CH_2_Cl_2_, N_2_, −5 °C; **III**: HCl/CH_3_OH, 65 °C.

DHPET was synthesized as depicted in Figure 4.

**Figure 5:**
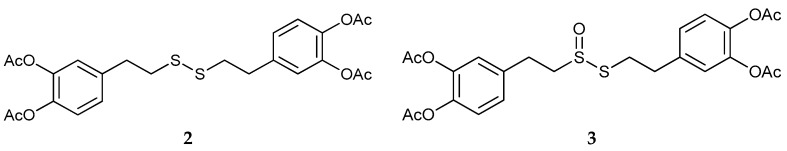
Proposed structures of main oxidation products from DHPET.

The autoxidation of DHPET in 1 mM bicarbonate buffer at pH 8.5 was monitored by UV-visible analysis. After generation of an initial colored intermediate identified as the *o*-quinone (absorption maximum at 480 nm), a brown insoluble precipitate was observed with almost complete loss of the catechol absorption. A similar behavior was observed with K_3_[Fe(CN)_6_]. However, attempts to reduce or acetylate the brown solid were unsuccessful. The oxidation of DHPET was then investigated at a lower pH using NaIO_4_ (2 eq) in phosphate buffer at pH 6.8, or (NH_4_)_2_[Ce(NO_3_)_6_] (2 eq) in phosphate buffer at pH 3 as the oxidants. In both cases, the analysis via thin layer chromatography (TLC) of the extractable organic fraction showed a significant amount of unreacted thiol after 2 h. When the oxidant was used in excess, no significant difference in product distribution was noticed (TLC evidence), probably because of the poor water solubility of the products determining their precipitation and the adsorption of the starting material. Complete substrate conversion was observed using horseradish peroxidase (HRP)/H_2_O_2_ as the oxidizing system. The TLC of the extractable organic fraction showed a distinct UV visible spot with no unreacted thiol left. Acetylation of the organic extract followed by purification of the resulting mixture by preparative layer cromatography (PLC) led to the isolation of two main fractions, which were analyzed by ^1^HNMR and ESI-Ion Trap mass spectrometry (Figures S7–S11), and were shown to consist of the disulfide of DHPET (**2**, Figure 5, left) and its oxygenated thiosulfinate derivative (**3**, Figure 5, right).

### 2.2.Synthesis and Oxidation of H_2_-DHBT (***7***)

**Figure 6:**
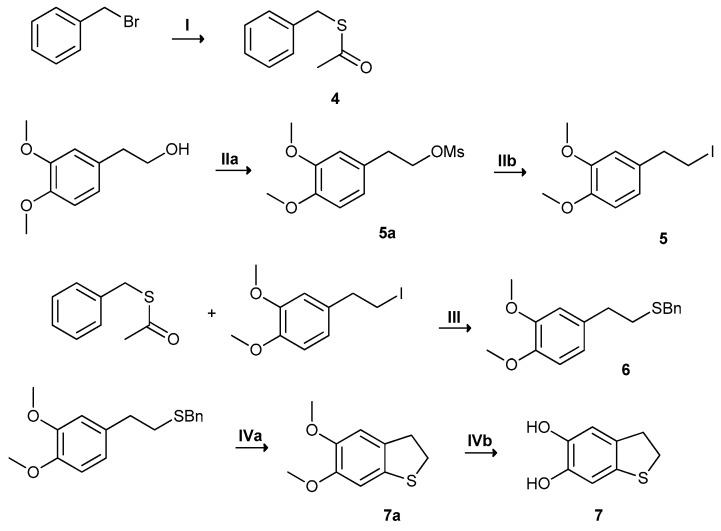
Synthetic scheme for 2,3-dihydro-5,6-dihydroxybenzo[b]thiophene (H_2_-DHBT, **7**). **I**: CH_3_COKS/(CH_3_)_2_CO, 56 °C; **IIa**: MsCl, Py/DCM, 0 °C; **IIb**: NaI/(CH_3_)_2_CO, 56 °C; **III**: NaOH/CH_3_OH, 0 °C; **IVa**: PIFA, BF_3_-Et_2_O/DCM, N_2_, −78 °C; **IVb**: BBr_3_/DCM, N_2_, 0 °C.

Since the oxidation of DHPET did not prove to be a viable entry to thiomelanin due to its failure to cyclize and polymerize, the synthesis of this thio-analog of eumelanin-type polymers was next pursued by synthesizing H_2_-DHBT, in order to bypass the difficult cyclization of DHPET quinone. Synthesis of H_2_-DHBT was achieved based on the scheme depicted in Figure 6 [29], its spectral characterization is provided in Figures S1 and S2.

Autoxidation of H_2_-DHBT in phosphate buffer at pH 8.8 over 24 h under stirring led to the formation of the aromatic derivative DHBT in 75% yield, while oxidation with potassium ferricyanide (2 eq) in 50 mM phosphate buffer pH 9.0 under an argon atmosphere for 30 min led to the same product in 90% yield by ethyl acetate extraction of the reaction mixture.

**Scheme 1:**
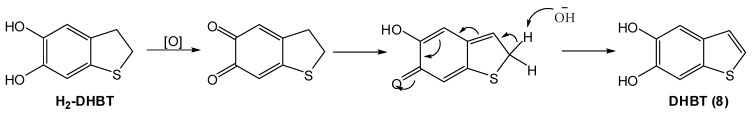
H_2_-DHBT proposed oxidation and isomerization steps to 5,6-dihydroxybenzothiophene (DHBT, **8**).

Spectrophotometric analysis of the oxidation of H_2_-DHBT with various oxidants indicated the rapid generation of a deep red coloration (absorption maximum around 490 nm), which slowly discharged due to conversion to the colorless DHBT (Figure S17). On this basis, it was concluded that oxidation of H_2_-DHBT leads initially to *o*-quinone, which undergoes two sequential isomerization steps based on proton shifts akin to those responsible for the isomerization of dopaminochrome to DHI [30] (Scheme 1). In support of this view, density functional theory (DFT) calculations at the TD-PBE0/6-311++G(2d,2p)/PCM level predicted for H_2_-DHBT quinone an absorption maximum of 485 nm, which is in very good agreement with the experimental value (Figure S18).

**Figure 7:**
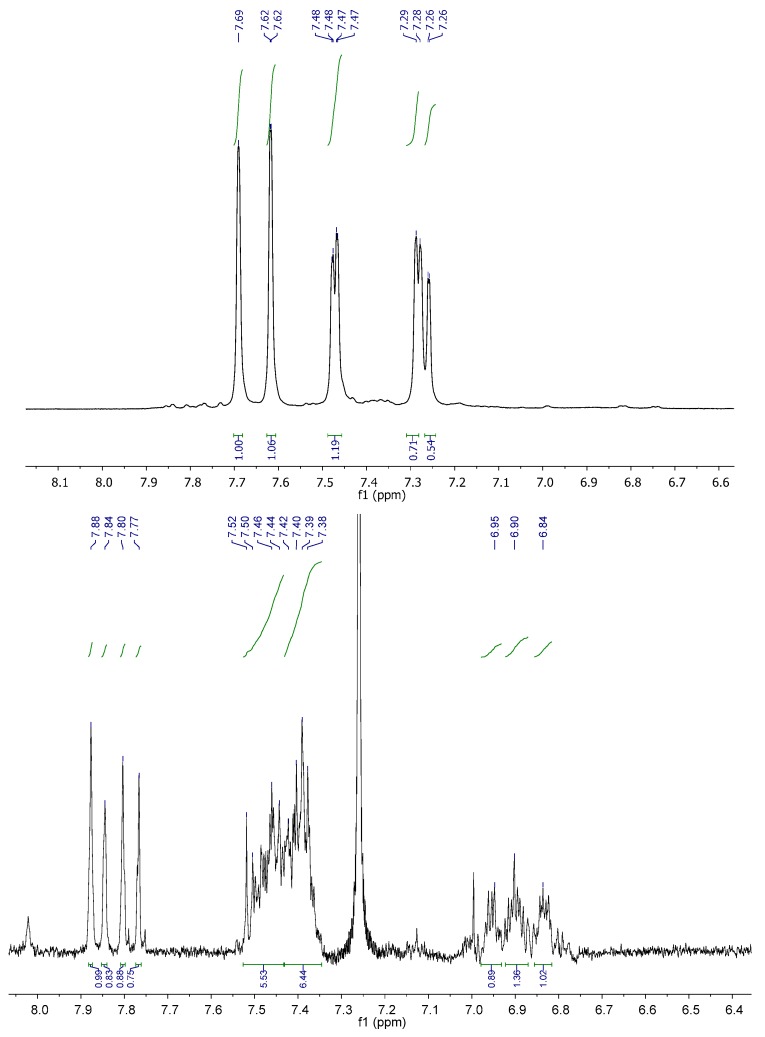
Comparison of the expansions (8.0–6.5 ppm) of the ^1^H NMR spectra in CDCl_3_ of DABT (**top**) and fraction III (**bottom**).

The spectral characterization of DHBT (**8**) and its acetylated derivative 5,6-diacetoxybenzo[b]thiophene (DABT) are reported respectively in Figures S3–S5 and Figure 7 (top), and its comparison with experimental data on DHI and its respective acetylated derivative, in Table S1. It is worth mentioning that the ca. 0.9 ppm downfield shift of the H-3 proton with respect to DHI denoted a markedly lower electron-releasing effect of sulfur relative to nitrogen. DFT simulation of the NMR spectrum (at the PBE0/6-311+G(d,p)/PCM level within the Gauge-Including Atomic Orbitals (GIAO) ansatz) gave data in quite good agreement with experimental data, and served as a benchmark for the subsequent spectral simulations at the oligomer level (Table S2).

### 2.3. Oxidative Polymerization of DHBT

Studies of the oxidative polymerization of DHBT were carried out using potassium ferricyanide or the horse radish peroxidase (HRP)/H_2_O_2_ system as the oxidant. With an excess of oxidant (e.g., 3 molar eq K_3_[Fe(CN)_6_]) centrifugation of the reaction mixture afforded an insoluble greyish-brown solid lighter in color than DHI melanin (Figure S19).

Scanning electron microscopy (SEM) images of thiomelanin showed aggregates of globular structures similar to DHI melanin and not exceeding 400 nm in diameter (Figure S20). LDI-MS analysis of the solid revealed the entire pattern of oligomeric species ranging from the dimer (*m/z* [M + Na]^+^ = 353) up to the decamer ([M + Na]^+^ = 1664), which denoted a substantial integrity of oligomer scaffolds (Figure S21). Inspection of each oligomer peak cluster indicated prevalently reduced catechol-type forms for the dominant species, in keeping with the relatively light polymer color. For example, the 680.96 *m/z* corresponds to the mass plus sodium of a fully-reduced tetramer.

**Figure 8:**
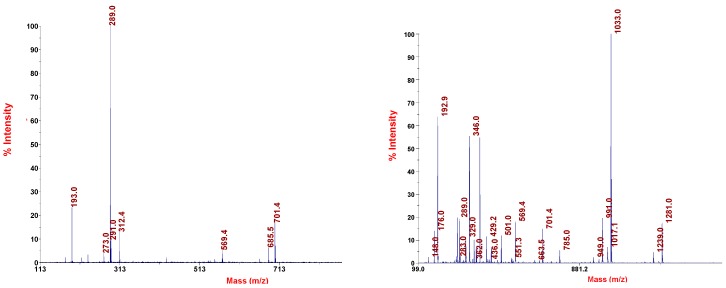
MALDI-MS spectra of 5,6-diacetoxybenzo[b]thiophene (DABT, **left**) and fraction III (mixture of acetylated tetramers of DHBT, **right**).

To inquire into the nature of the low oligomer intermediates of thiomelanin, a typical oxidation mixture was halted in the early stages by reductive treatment with sodium dithionite, and extracted with ethyl acetate. The organic fraction (ca. 60% *w*/*w* based on initial DHBT) was desiccated, acetylated, and the resulting mixture was analyzed by TLC and HPLC by a protocol similar to that reported for the identification of DHI oligomers (Figure S12). Unfortunately, despite a careful search for optimized reaction times and conditions, in no case was it possible to extract more than trace amounts of oligomer intermediates, which prevented a complete and detailed characterization, e.g., by isolation and purification of single products. Nonetheless, by repeating the oxidation protocol three times and by chromatographing the combined acetylated extracts on silica, it was possible to obtain three fractions, denoted I–III, which were analyzed by ^1^HNMR and MALDI-MS in comparison with the spectral characterization of DABT (Figure 7 (top), Figure 8 (left), and Figure S5). Fraction I (less than 4% *w*/*w* yield based on reacted monomer) gave an intense pseudomolecular ion peak at *m/z* 537 [M + K]^+^, which suggested a dimer (Figure S13). Its ^1^H-NMR spectrum displayed three signals: a singlet at δ 7.8 and two doublets for the coupled thiophene protons, one of which experienced a marked shielding effect shifting resonance up to δ 6.7, indicating a symmetric structure (Figure S14). Although it was not possible to record meaningful heterocorrelated NMR spectra, based on the upfield shift of the H-3 proton and the excellent agreement with the DFT-simulated ^1^H-NMR spectrum, it was possible to identify the compound as the acetyl derivative of the symmetric 4,4′-dimer (Table S2).

Fractions II and III (8 and 2% *w*/*w* respectively) showed on MS analysis base peaks consistent with trimers and tetramers, respectively (Figure S15 and Figure 8 (right)). Both fractions displayed in the ^1^H-NMR spectra three distinct sets of signals at δ 7.7–7.9 (a), 7.3–7.5 (b), and 6.7–7.0 (c), which suggested intimate mixtures of oligomers reflecting apparently regular patterns of polymerization (Figure S16 and Figure 7 (bottom)). The observed complexity in the spectra of the acetylated oligomer fractions, e.g., the trimer-based one, may be due in part to minor components derived from incomplete acetylation, as suggested by mass spectrometric analysis, as well as to anisotropism, as reported in the case of 5,6-dihydroxyindole-2-carboxylic acid [31].

**Figure 9:**
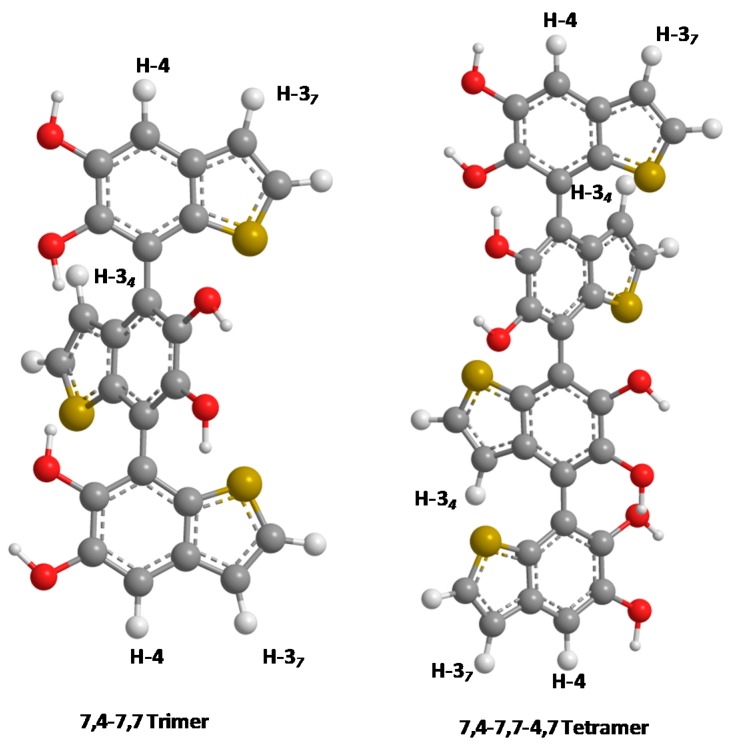
Ball and stick models of representative trimer and tetramer structures exemplifying the case of 7-linked terminal units in which only inner units contribute to H-3 protons of set c (lowest signal area ratio (set c):(set b) = 1:5 for the trimer and 2:6 = 1:3 for the tetramer).

Although the exceedingly low amounts available and the similar chromatographic behavior frustrated all attempts at separating the oligomer components of the trimer and tetramer mixtures, nor could we succeed in recording meaningful correlated NMR spectra, it was possible nonetheless to gain significant insights into the general structural features of the main components of each fraction by careful scrutiny of the ^1^H-NMR spectra. Taking the 4,4′-dimer as a reference, it was possible to assign set a to H-4/H-7 protons, set b to H-2/H-3_7_ protons, and set c to H-3_4_ protons, (where “H-3_7_”refers to H-3 protons on terminal 7-linked units, and “H-3_4_”to H-3 protons on inner or terminal 4-linked units, Figure 9). Moreover, determination of the relative areas of set a vs. sets b + c in the trimer and tetramer series gave (H-4 + H-7)/(H-2 + H-3) ratios of ca. 29% and 22%, respectively, which matched fairly well with the theoretical relative areas of 33% and 25% expected for mixtures of linear trimers and tetramers, respectively.

It was further argued that the (set c)/(set b) (i.e., H-3_4_/{H-3_7_ + H-2}) area ratio in the trimer and tetramer series could provide information about the proportion of 4-linked versus 7-linked terminal units. Since all inner units (i.e., *n* − 2 units) are 4-linked, and thus contribute *n* − 2 protons to signal set c for any oligomer with *n* units, the area ratio would vary from (*n* − 2)/(*n* + 2) (with two 7-linked terminal units) to *n*/*n* = 1 (with two 4-linked terminal units, 100% of 4-linked units). Thus, for trimers (*n* = 3), the lowest (set c)/(set b) area ratio with two 7-linked terminal units would be 0.20, while for tetramers (*n* = 4), the lowest (set c)/(set b) area ratio with two 7-linked terminal units would be 0.33. More generally, denoting as *ρ* the ratio between (set c)/(set b), it follows that:

$$\rho =\frac{n-x}{n+x}$$

where *x* is the number of 7-linked terminal units out of *n* total units. Since *x* ranges between 0 and 2, the percentage of 7-linked terminal units is given by 100 *x*/2. Rearranging the equation, we then get:

$$x=n\cdot \frac{1-\rho }{1+\rho }$$

Based on the experimental ratios determined for fractions II and III (trimers and tetramers), i.e., 0.62 and 0.56, respectively, it was possible to estimate that, on average, trimer and tetramer populations contained 35% and 56% of 7-linked terminal units. These data are not sufficient to draw any general conclusion about the relative reactivity of DHBT at the 4- versus 7-positions. Although fraction I is the only one where a dimer could be isolated, and it shows essentially a 4,4′-linked dimer, this would not rule out the possibility that 4,7′- and/or 7,7′-linked dimers are also formed and involved in oligomer growth. However, it is worth noting that the 35% of the 7-linked terminal units determined in the trimer fraction would be consistent with a main origin from the isolated 4,4′-dimer. This latter would consistently give rise to only two isomeric trimers, featuring either one or no 7′-linked terminal units, i.e., 25% of total terminal units on average in case of statistical formation of the two trimers. In any case, the lower solubility/extractability or higher reactivity may be an explanation for missing putative 4,7′- and 7,7′-linked dimers. Likewise, it is possible that oligomers featuring other modes of coupling, e.g., via the 2-position, are also formed.

### 2.4. Mechanistic Issues and Density Functional Theory (DFT) Calculations

The structural characterization of the oligomer intermediates in the oxidative conversion of DHBT to thiomelanin revealed an unexpectedly regioregular polymerization process involving the carbocyclic ring. This is in marked contrast with the typical reactivity of the DHI system, dominated by 2,4′- and 2,7′-couplings. To inquire into the factors underlying the divergent oxidative behavior of DHBT and DHI, a detailed computational investigation of DHBT and its semiquinone and quinine was carried out at the DFT level of theory. One-electron oxidation of DHBT is predicted to involve preferentially the C6-OH group, leading to (5-hydroxy-1-benzothiophen-6-yl)oxidanyl; the isomeric (6-hydroxy-1-benzothiophen-5-yl)oxidanyl (in water) is about 1.9 kcal mol^−1^ higher in free energy. This is rather similar to the situation of the corresponding DHI-derived radicals, which are separated from each other by ca. 1.6 kcal mol^−1^. However, deprotonation of the DHBT radical is significantly easier than in DHI, with a p*K*_a_ difference reaching ca. 1.5 units, as estimated by comparison of the relevant Δ*G*_SMD,RRHO_ values with those of a number of reference acid/base pairs. Thus, the DHBT radical is predicted to exist predominantly in the anionic form, even in neutral aqueous media. On the assumption that the coupling of one-electron oxidized forms may represent a crucial initial step in the oxidative polymerization of DHBT [32], Table S3 reports computed spin densities on selected positions of the DHBT semiquinone, in its neutral and anionic form. For comparison, values obtained at the same level for DHI are also reported.

In the anionic form of DHBT semiquinone, the highest spin density is on C2, with lower but significant values on C3 and on C7. By contrast, the anionic form of DHI semiquinone has high spin density on C2 and on C3, and almost no density on C4/C7. Among the neutral semiquinones, the preferred C6-O centered radical has the highest spin density on C7 in DHBT, and on C2 in DHI. In all cases, spin density on C4 is significantly higher in DHBT-derived radicals than in their DHI-derived counterparts. This could point to a high reactivity of monomeric DHBT towards coupling through the 4- and 7-position. However, the reasons of a reduced involvement of C2 are less clear.

**Scheme 2:**
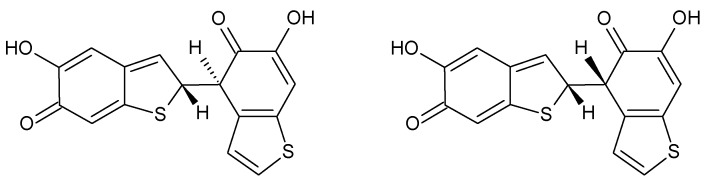
Initial product generated from the 2,4′-coupling of a neutral 6-yl DHBT semiquinone with a neutral 5-yl DHBT semiquinone. Two diastereoisomeric forms, (2R, 4′R) and (2R, 4′S), are shown.

Additional insights into the coupling modes of DHBT were sought by examination of the first-formed dimers (see e.g., Scheme 2). These intermediates are expected to undergo facile and irreversible rearomatization to the corresponding bi-benzothiophene products.

A full conformational exploration (including two diastereoisomeric forms of each dimer, different initial conformations of the six-membered rings involved in binding, and at least three rotamers around the interring bond) was carried out on all possible dimers arising from the coupling of two neutral DHBT semiquinones (Table 1), or from the coupling of one neutral semiquinone with one semiquinone in anionic form (Table S4). Coupling of two anionic semiquinones should be disfavored by coulombic repulsions, and was disregarded. Empirical dispersion corrections [33] were employed in this series of calculations, in order to better reproduce the influence of interring interactions. For each different positional isomer, the relative free energy of the most stable form was identified. Complete data on all diastereoisomers/conformers are provided in Tables S5 and S6, respectively.

In the neutral state (Table 1), as well as in the anion state (Table S4), the most stable structure corresponds to a 4,4′-coupling dimer; alternative coupling modes are energetically disfavored. The difference is appreciable for the neutral dimers (the second-best structure being the 4,7′-dimer at ca. 1.0 kcal mol^−1^), and much less significant for the monoanionic dimers. To the extent that the stability of the products can be used to infer information on the corresponding transition states (Hammond’s postulate), this could be taken as an indication of the tendency of the DHBT semiquinones to undergo preferential 4,4′-coupling. In principle, further insights into the selectivity of the reaction could be gained by systematic identification of all of the alternative transitions structures; on account of the substantial computational effort required, this approach will be the object of a separate and more general reexamination of catechol coupling mechanisms.

### 2.5. General Properties of Thiomelanin

**Figure 10:**
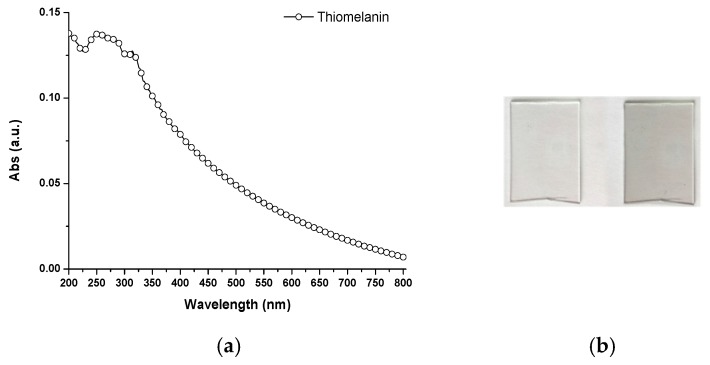
(**a**) UV-visible spectrum of a thin film of thiomelanin on quartz; (**b**) Picture of the quartz sleeve before and after dip coating with thiomelanin.

To gain a preliminary insight into the DHBT polymer (thiomelanin), its spectral properties were investigated in comparison with those of DHI melanin and DHICA melanin. To determine the UV-visible absorption properties of thiomelanin without contributing scattering effects, thin films of thiomelanin were prepared by the dip-coating methodology from carbonate buffer pH 9 (Figure 10b). Under the same conditions, DHI polymerization did not result in detectable films [22], which pointed to noticeable adhesion properties of thiomelanin compared with DHI melanin.

Data in Figure 10a indicate limited absorption in the visible region with no distinct band, on account of the light coloration. Interestingly, in an aqueous suspension, thiomelanin gave a spectrum with a distinct band/shoulder in the UV region, around 300 nm (Figure S22). This feature was akin to that of the absorption spectrum of DHICA melanin [6], and suggested a high proportion of reduced units unavailable to conjugation and electron delocalization.

The attenuated total reflection-fourier transform infrared (ATR/FT-IR) spectrum of thiomelanin (Figure S23) An intense large band in the oxydril group stretching region (3500 cm^−1^) was apparent, along with distinct bands around 1650, 1350, and 1250 cm^−1^ attributable to the C = C stretching of the aromatic skeleton.

**Figure 11:**
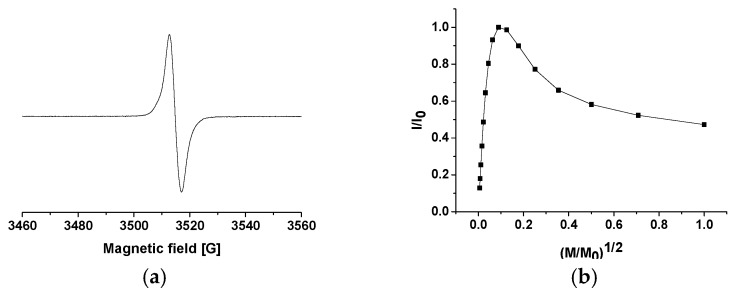
Electron Paramagnetic Resonance (EPR) signal (**a**) and power saturation profile (**b**) of thiomelanin.

Figure 11a shows the electron paramagnetic resonance (EPR) spectrum of thiomelanin prepared by ferricyanide oxidation; spectral parameters are collected in Table 2, where they are also compared with data obtained for DHI and DHICA melanins.

Thiomelanin was found to exhibit a typical eumelanin-like signal: a structure-less singlet at a *g* value due likely to the presence of carbon-centered radicals. The spin density was of the same order of magnitude of that of DHICA melanin, both being at least 10-fold lower than that estimated for DHI melanin. Considering that the stabilization of unpaired electrons is connected to their delocalization, these observations point to a less extended conjugation of π-electron systems in thiomelanin (as in DHICA melanin) with respect to DHI melanin. The thiomelanin signal line width, defined as the peak-to-peak distance, was very close to that of DHICA melanin, and significantly lower than that observed for DHI melanin. This could be, at least partially, ascribed to the lower spin density: broadening of the EPR line could result from the dipolar interaction of unpaired magnetic moments of free radicals located at a short distance, and consequently is expected to increase with the spin density. Thus, the data would overall concur to support for thiomelanin a lower degree of chemical/structural disorder with respect to DHI melanin. This conclusion was confirmed by the power saturation curve of thiomelanin (Figure 11b), which decreases at high microwave power, indicating that radicals are homogeneously located in the sample on similar aromatic groups. This curve is different from that of DHI melanin, which is typical of heterogeneous radical distribution, but closer to that of DHICA melanin, in which monomer units are linked through 4,4′-bonds.

## 3. Materials and Methods

All reagents were commercial and were used as purchased. Organic solvents were used as purchased. Water was of MilliQ^®^ quality (Merck Millipore, Billerica, MA, USA). Buffers were prepared by standard procedures. A Crison pH-meter equipped with a 5014 Crison electrode (Crison Instruments, Barcelona, Spain) was used for pH measurements at room temperature.

UV-Vis absorption spectra were registered at room temperature on a V-560 JASCO spectrophotometer (Jasco Inc, Easton, MD, USA) using calibrated 2 mL cuvettes and, for solid state samples, on a Cary 4000 UV-Vis spectrophotometer (Agilent Technologies, Santa Clara, CA, USA) using the solid sample holder. NMR spectra were recorded at 400 MHz in deuterated solvents on a Bruker DRX 400 or Bruker Avance DRX 400 (Bruker, Billerica, MA, USA) and at 500 MHz on a Varian Inova 500 (Varian, Palo Alto, CA, USA), δ values are reported in ppm, and coupling constants are given in Hz.

Resonance assignments follows from analysis of ^1^H,^1^H COSY (^1^HNMR) and ^1^H,^13^C HSQC and HMBC (^13^C NMR) spectra. HPLC analyses for reaction monitoring were performed on an Agilent 1100 series instrument equipped with an LC-10AD VP pump and a G1314A UV-Vis detector using a Sphereclone C_18_ column (4.6 mm × 150 mm, 5 μm). LC-MS analysis was conducted on an electrospray ionization-time of flight (ESI-TOF) spectrometer 1260/6230DA Agilent Technologies in positive ion mode. IR spectra were run on a Nicolet 5700 FT-IR + Smart performer spectrometer mounting a Continuum FT-IR Microscope (Thermo Fisher Scientific, Waltham, MA, USA) and on Bruker Optics TENSOR 27 FT-IR. SEM. Electron paramagnetic resonance (EPR) spectra were obtained by a previously set procedure [6]. Melanin samples were measured using an X-band (9 GHz) Bruker Elexys E-500 spectrometer (Bruker, Rheinstetten, Germany), equipped with a high sensitivity probe head. Polymer powder was transferred to a flame-sealed glass capillary which, in turn, was coaxially inserted in a standard 4 mm quartz sample tube. Measurements were carried out at room temperature with the following settings: sweep width, 100 G; resolution, 1024 points; modulation frequency, 100 kHz; modulation amplitude, 2.0 G. Preventively, it was verified that the amplitude of field modulation was low enough to avoid detectable signal overmodulation. Microwave power was ~4 mW to avoid saturation of the resonance absorption curve. To improve the signal-to-noise ratio, a total of 64 scans was accumulated. For power saturation experiments, microwave power was increased from 0.004 to 64 mW. To evaluate g value and spin density, Mn^2+^-doped MgO were used as an internal standard [34]. Since sample hydration was not controlled during the measurements, spin density values had to be considered as order of magnitude estimates [35]. MALDI analyses were run on an AB Sciex TOF/TOF 5800 instrument (Sciex, Framingham, MA, USA) using 2,5-dihydroxybenzoic acid as the matrix. Insoluble samples, such as those of thiomelanin, were applied to the plate from a fine suspension in ethanol obtained by homogenization in a glass-to-glass potter. Spectra represent the sum of 15,000 laser pulses from randomly chosen spots per sample position. Raw data are analyzed using the computer software provided by the manufacturers, and are reported as monoisotopic masses. DFT calculations were carried out with the Gaussian package of programs (Gaussian Inc., Carnegie Mellon University Pittsburgh, PA, USA) [36]. All structures were geometry optimized at the DFT level, with the hybrid functional PBE0 [37] and a reasonably large basis set [6-31+G(d,p)]. For comparison, single-point DFT calculations were also carried out with the hybrid meta-GGA functional M06-2X [38]. For each species, different tautomers/conformers and different protonation states were explored. In those cases where conformational enantiomers exist, a single enantiomeric series has been explored. Computations were performed either in vacuo, or using a polarizable continuum medium (PCM) [39,40,41,42]. Due to the faster convergence, a scaled van der Waals cavity based on universal force field (UFF) radii [43] was used, and polarization charges were modeled by spherical Gaussian functions [44,45]. Non-electrostatic contributions to the solvation free energy were disregarded at this stage, as they were accounted for in single-point PCM calculations employing radii and non-electrostatic terms of the solvation model based on density (SMD) [46]. Vibrational-rotational contributions to the free energy were also computed. Empirical dispersion corrections were introduced using Grimme’s D3 version with Becke–Johnson damping [33].

UV-Vis spectra of the main species were computed in vacuo or in solution using the time-dependent density functional theory (TD-DFT) approach [47,48,49,50,51], with the PBE0 functional and the 6-311++G(2d,2p) basis set. To produce graphs, transitions below 5.6 eV were selected, and an arbitrary Gaussian line width of 0.25 eV was imposed; the spectra were finally converted to a wavelength scale.

NMR shielding tensors were computed within the gauge-including atomic orbitals (GIAO) ansatz [52,53] with the PBE0 functional and the 6-311+G(d,p) basis set. Computed isotropic shieldings were converted into chemical shifts using values obtained at the same level for benzene in chloroform (PCM) as reference [54].

## 4. Conclusions

### Eumelanin Versus Thiomelanin: the Role of Nitrogen and Opportunities from Replacement by Sulfur

In conclusion, the results of this study have furnished an unprecedented demonstration that nitrogen is central to most of eumelanin’s physical and chemical properties, and that its replacement by sulfur may a valuable entry to an entirely novel class of melanin-related materials of potential practical interest.

More in detail, several important conclusions about the role of sulfur in eumelanin synthesis and properties could be drawn:

1. Replacement of nitrogen by sulfur in dopamine completely precludes the formation of eumelanin-type polymers due to the inability of DHPET to undergo intramolecular cyclization. However, the observed conversion of DHPET into its disulfide and related oxygenation products may be of interest for the development of novel antioxidant systems responding to oxidizing stimuli via both the catechol and thiol/disulfide groups.
2. Oxidation of H_2_-DHBT proceeds via a red-orange chromophoric phase that displays an absorption maximum similar to that of dopaminochrome. This chromophore was attributed to the corresponding quinone, i.e., the sulfur analog of dopaminochrome, on the basis of DFT simulations. The analogies both in the chromophore and the tendency to rearrangement would apparently rule out a specific role of nitrogen in aminochrome chemistry and properties.
3. DHBT appear to be more difficult to oxidize than DHI, which makes reduced species predominate (unless subject to strong oxidizing environments), and radical species less abundant.
4. Thiomelanin appears to be markedly different from DHI melanin in a number of properties, including visible light absorption, redox potential, and especially, adhesion to quartz and glass surfaces by dip coating.

Taken together, these results provide evidence that replacing nitrogen by sulfur imparts regioregularity to eumelanin structure through modifying visible absorption properties and the average redox state. Hence, nitrogen is a main determinant of structural disorder in eumelanins, including the prevalent coupling of DHI via the 2-position, and accounts for strong visible light absorption and the dark colors of the polymers. These properties reflect the tendency of DHI to couple at the 2-position adjacent to the heteroatom, a behavior that is apparently hindered by sulfur. The main consequence is that freely-rotating 2-linked DHI units can adopt planar conformations enabling extensive electron delocalization in oxidized quinonoid structures, while 4- and 7-linked DHBT units cannot, due presumably to the bulky sulfur atom. This would explain why thiomelanin has limited visible absorption, which is also probably a consequence of poor packing interactions in atropisomeric oligomer mixtures, and a prevalently reduced state (MALDI MS evidence). Similar arguments have been proposed to explain the difference between DHI and DHICA melanins [6].

The potential of the heteroatom replacement approach reported herein deserves further attention to inquire into eumelanin structure–property relationships, and to design novel melanin-type materials with tailored properties for surface functionalization and coating. This latter aspect is the focus of ongoing work in our laboratories.

## Figures and Tables

**Table 1:** Relative stabilities of different positional isomers of the first-formed dimeric products arising from coupling of two neutral DHBT semiquinones.

Dimer	Coupling Mode	ΔG_SMD,RRHO_ (kcal mol^−1^)
2,2′	2 of 6-yl semiquinone on 2 of 6-yl semiquinone	0.99
2,4′	2 of 6-yl semiquinone on 4 of 5-yl semiquinone	1.08
2,7′	2 of 6-yl semiquinone on 7 of 6-yl semiquinone	1.64
4,4′	4 of 5-yl semiquinone on 4 of 5-yl semiquinone	0.00
4,7′	4 of 5-yl semiquinone on 7 of 6-yl semiquinone	0.93
7,7′	7 of 6-yl semiquinone on 7 of 6-yl semiquinone	2.53

**Table 2:** Electron paramagnetic resonance (EPR) values for 5,6-dihydroxybenzo[b]thiophene (DHBT), 5,6-dihydroxyindole carboxylic acid (DHICA) and 5,6-dihydroxyindole (DHI) melanins.

Melanin	*g* (±0.0003)	ΔB [G] (±0.05 G)	Spin Density (spin/g)
Thiomelanin	2.0032	4.4	4 × 10^17^
DHICA mel ^1^	2.0034	4.4	5 × 10^17^
DHI mel ^1^	2.0035	5.6	4 × 10^18^

^1^ Ref. [6].

## Supplementary Materials

Supplementary materials can be found at www.mdpi.com/1422-0067/18/10/2169/s1.

## Abbreviations

DHBT	5,6-dihydroxybenzo[b]thiophene
DHI	5,6-dihydroxyindole
DHICA	5,6-dihydroxyindole-2-carboxylic acid
PDA	Polydopamine
DHPET	2-(3,4-dihydroxyphenyl)ethanethiol
H_2_-DHBT	2,3-dihydro-5,6-dihydroxybenzo[b]thiophene
DABT	5,6-diacetoxybenzo[b]thiophene
DAI	5,6-diacetoxyindole
