# Supplementary material for: Replacing Nitrogen by Sulfur: From Structurally Disordered Eumelanins to Regioregular Thiomelanin Polymers
Source: Int J Mol Sci. 2017 Oct 17;18(10):2169. doi: 10.3390/ijms18102169 (PMC5666850; doi:10.3390/ijms18102169)
Supplement: Supplementary file 1 [file ijms-18-02169-s001.pdf]

SUPPORTING INFORMATION

Replacing Nitrogen by Sulfur: From Structurally Disordered Eumelanins to Regioregular Thiomelanin Polymers

Mariagrazia Iacomino [1], Juan Mancebo-Aracil [2], Mireia Guardingo [2,3], Raquel Martín [3], Gerardino D'Errico[1], Marco Perfetti [1], Paola Manini [1], Orlando Crescenzi [1], Félix Busqué [3], Alessandra Napolitano [1], Marco d'Ischia [1,*], Josep Sedó [2] and Daniel Ruiz-Molina [2,*]

[1] Department of Chemical Sciences, University of Naples "Federico II", Via Cintia 4, I-80126, Naples, Italy;
[2] Catalan Institute of Nanoscience and Nanotechnology (ICN2), CSIC and The Barcelona Institute of Science and Technology, Campus UAB, 08193 Bellaterra, Spain
[3] Department de Química, Universitat Autònoma de Barcelona (UAB), Campus UAB, 08193 Bellaterra, Spain
* dischia@unina.it; dani.ruiz@icn2.cat;

Synthesis and structural characterization of 3,4-dihydroxyphenyl ethanethiol (DHPET, 1). *Step I, Synthesis of S-3,4-dimethoxyphenethyl ethanethioate (***1a***).* To a solution of 2-(3,4 – dimetoxyphenyl)ethanol (1.5 g, 8.24 mmol) in dry tetrahydrofuran (THF, 42 mL) methanesulfonyl chloride (MsCl, 1.55 mL, 21.3 mmol) and triethylamine (Et_3N, 2.51 mL) were added dropwise under N_2 atmosphere. The reaction mixture was stirred at r.t. for 2 h. Potassium thioacetate (3 g, 26.3 mmol) was dissolved in dry DMF (36 mL). The latter solution was slowly added to the reaction mixture. After 4 h, the solvent was evaporated under reduced pressure and extracted (water/ethyl acetate), the combined organic layers were dried over anhydrous Na_2SO_4, evaporated and purified by flash silica chromatography (hexane:ethyl acetate 9:1). The desired product (**1a**) was obtained as a brown oil (1.72 g, 87 % yield).

(**1a**): ^{1}H NMR (400 MHz, $CDCl_3$) δ 6.80 (d, *J* = 8.1 Hz, H-6), 6.77 (s, H-2), 6.73 (d, *J* = 8.0 Hz, H-5), 3.88 and 3.86 (s, H-11, H-12), 3.10 (t, *J* = 7.7 Hz, H-8), 2.81 (t, *J* = 7.7 Hz, H-7), 2.33 (s, H-10). ^{13}C NMR (91 MHz, $CDCl_3$) δ 193.2 (C-9), 149.0 (C-3), 148.0 (C-4), 133.0 (C-1), 122.0 (C-6), 115.2 (C-5), 112.1 (C-2), 56.1 and 55.8 (C-11, C-12) 36.1 (C-8), 33.2 (C-7), 30.3 (C-10).

*Step II, Synthesis of S-3,4-dihydroxyphenethyl ethanethioate (***1b***).* To a solution of **1a** (1.53 g, 5.88 mmol) in CH_2Cl_2 (24 mL) a 1 M solution of BBr_3 in CH_2Cl_2 (32 mL) was slowly added under N_2 atmosphere at -5 °C. After 6 h, the reaction mixture was extracted with water. The combined organic layers were dried over anhydrous Na_2SO_4 and evaporated to give **1b** (1.0 g, yield >> 99%). No further purification was needed.

(**1b**): ^{1}H NMR (400 MHz, $CDCl_3$) δ 6.82 (d, *J* = 8.0 Hz, H-6), 6.73 (s, H-2) 6.65 (d, *J* = 8.0 Hz, H-5), 2.82 (d, *J* = 6.6 Hz, H-7), 2.79 (d, *J* = 6.6 Hz, H-8), 2.07 (s, H-10). ^{13}C NMR (91 MHz, $CDCl_3$) δ 193.2 (C-9), 145.0 (C-3), 144.0 (C-4), 133.0 (C-1), 122.0 (C-6), 116.2 (C-5), 115.4 (C-2), 36.1 (C-8), 33.6 (C-7), 30.5 (C-10).

*Step III, Synthesis of S-3,4-dihydroxyphenyl ethanethiol (***DHPET, 1***).* To a solution of **1b** (1.0 g, 5.85 mmol) in CH_3OH (90 mL) 12 M HCl was added (50 drops). The mixture was left under stirring overnight under reflux. After 12 h, the solvent was evaporated under

reduced pressure and the reaction extracted by water/ethyl acetate. DHPET was recovered without further purification as a brown oil (0.9 g, yield = 90%).

(**1**): ^{1}H NMR (360 MHz, $CDCl_3$) δ 6.80 (d, *J* = 8.0 Hz, H-6), 6.77 (s, H-2), 6.73 (d, *J* = 8.0 Hz, H-5), 2.98 (t, *J* = 6.8 Hz, H-7), 2.85 (t, *J* = 6.8 Hz, H-8). ^{13}C NMR (91 MHz, $CDCl_3$) δ 149.0 (C-3), 148.1 (C-4), 131.0 (C-1), 121.0 (C-6), 120.9 (C-2), 112.2 (C-5), 38.7 (C-7), 35.2 (C-8).

Synthesis and structural characterization of 5,6-dihydrobenzo[b]thiophene (DHBT, 8).

*Step I, Synthesis of S-benzyl ethanethioate (***4***).* To a solution of benzyl bromide (1.26 g, 7.4mmol) in Acetone (20 mL) potassium thioacetate (0.9 g, 7.88 mmol) was added . The reaction mixture was heated at reflux for 2h, evaporated and extracted with ethyl acetate, the combined organic layers were dried over anhydrous Na_2SO_4 and evaporated, affording **4** as a colourless oil (1.20 g, 99%).

(**4**): ^{1}H NMR (360 MHz, CD_3Cl) δ 7.29 (m, protoni aromatici), 4.12 (s, H-5), 2.35 (s, H-7). ^{13}C NMR (91 MHz, $CDCl_3$) δ 137.57 (C-1), 128.80 (C-3,3′), 128.63 (C-2,2′), 127.27 (C-4), 33.46 (C-7), 30.34 (C-5).

*Step IIa and IIb, Synthesis of 2-iodoethyl-3,4-dimethoxybenzene (***5***).* To a solution of 2-(3,4 – dimetoxyphenyl)ethanol (1.5 g, 8.24 mmol) in CH_2Cl_2 (20 mL) at 0 °C pyridine (0.73 mL, 9.06 mmol) and methanesulfonyl chloride (0.66 mL, 9.06 mmol) were added. The reaction mixture was stirred at rt for 12 h, poured into 5% aqueous HCl (12 mL), and extracted with CH_2Cl_2, the combined organic layer was dried over anhydrous Na_2SO_4, evaporated and used without further purification for the next step of the synthesis (yield: 85%).

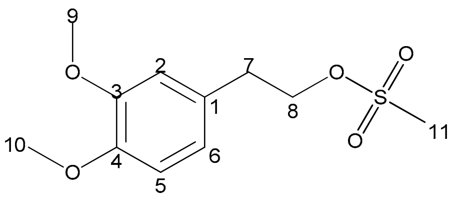

5a

^{1}H NMR (360 MHz, $CDCl_3$) δ 6.80 (d, J = 8.0 Hz, H-6), 6.77 (s, H-2), 6.73 (s, H-5), 4.37 (t, J = 6.9 Hz, H-8), 3.86 (s, H-10), 3.84 (s,H-9), 2.98 (t, J = 6.8 Hz, H-7), 2.85 (s, H-11).^{13}C NMR (91

MHz, $CDCl_3$) δ 149.0 (C-3), 148.0 (C-4), 131.0 (C-1), 121.0 (C-6), 112.2 (C-5), 112.1 (C-2), 70.52 (C-8), 55.9 (C-10), 55.8 (C-9), 37.3 (C-11), 35.2 (C-7).

Subsequently, to a solution of **5a** (2.0 g, 7.83mmol) in acetone, NaI (1.76 g, 11.7 mmol) was added. The mixture was heated at reflux overnight, cooled, and evaporated. The oily mixture was then extracted with water/CH_2Cl_2, the combined organic layers were dried over anhydrous Na_2SO_4 and evaporated. Flash chromatography of the crude mixture, with Hexane: Ethyl Acetate 9:1 as eluting system, afforded 1.39 g (61%) of **5** as a pale yellow oil.

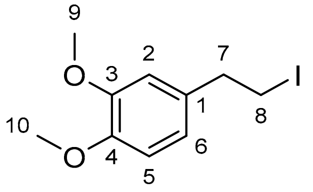

5

^{1}H NMR (360 MHz, $CDCl_3$) δ 6.8 (d, J = 8.1 Hz, H-6), 6.75 (s, H-2) 6.6 (d, J = 8.1 Hz, H-5), 3.9 (s, H-10), 3.8 (s, H-9), 3.3 (t, J = 7.8 Hz, H-8), 3.1 (t, J = 7.8 Hz, H-7). ^{13}C NMR (91 MHz, $CDCl_3$) δ 148.9 (C-3), 147.9 (C-4), 133.3 (C-1), 120.4 (C-6), 111.6 (C-2), 111.3 (C-5), 55.9 (C-9,10), 40.0 (C-8), 6.2 (C-7).

*Step III, Synthesis of benzyl(3,4-dimethoxyphenethyl)sulfane (**6**).* To a solution of **5** (1.2 g, 4.1 mmol) in MeOH (20 mL) at 0 °C, 0.5 M NaOH in MeOH (10 mL) was slowly added and then a solution of **4** (0.68 g, 4.1 mmol) in MeOH (10 mL). The reaction mixture was stirred at room temperature for 24 h, poured into water, and extracted with CH_2Cl_2, and the combined organic layers were dried over anhydrous Na_2SO_4 and evaporated. Flash chromatography of the crude mixture with Hexane: Ethyl Acetate 9:1 as eluting system, afforded 0.98 g (85%) of **6** as a colorless oil.

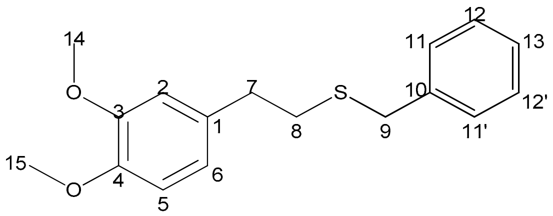

6

*Step IVa and IV, Synthesis of 2,3-dihydro-5,6-dihyroxybenzothiophene (***7, H_2-DHBT***).* A DCM (66 mL) mixture of PIFA and BF_3-Et_2O was added dropwise to a solution of **6** (0.95 g, 3.3 momol) in DCM (95 mL) at -78 °C under N_2. The reaction was stirred for 20 minutes, then quenched with $MeNH_2$ (40%), stirred for 30 minutes and acidified with HCl 10% until pH 6.5. The mixture was extracted with CH_2Cl_2 and purified by flash chromatography using Hexane : Ethyl acetate 9:1 as eluting system affording to 0.4 g (63%) of 5,6-dimethoxy-2,3-dihydrobenzo[b]thiophene (**7a**) as a white solid.

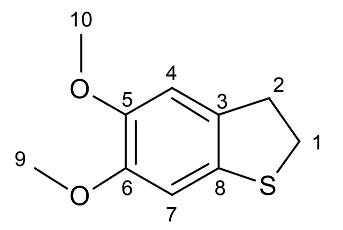

7a

^{1}H NMR (360 MHz, $CDCl_3$) δ 6.8 (s, H-7), 6.7 (s, H-4), 3.84 (s, H-9), 3.83 (s, H-10), 3.4 (t, J = 7.7 Hz, H-2), 3.2 (t, J = 7.7 Hz, H-1). ^{13}C NMR (91 MHz, $CDCl_3$) δ 148.84 (C-6), 146.64 (C-5), 132.29 (C-3), 131.57 (C-8), 108.73 (C-4), 105.86 (C-7), 56.39 (H-11), 56.23 (H-10), 36.50 (C-1), 34.28 (C-2).

In the next reaction, BBr_3 (5 mL) was added dropwise to a solution of **7a** (0.17 g, 0.89 mmol) in DCM (4 mL), at 0 °C and under N_2 atmosphere. After 90 minutes, the reaction mixture was evaporated and extracted with H_2O/Diethyl Ether affording to 0.13 mg (90%) of **7** as a white solid.

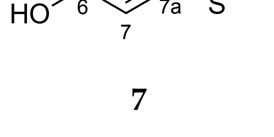

7

^{1}H NMR (360 MHz, CD_3OD) δ 6.66 (s, H-7), 6.58 (s, H-4), 3.27 (t, J = 7.7 Hz, H-3), 3.08 (t, J = 7.6 Hz, H-2); ^{13}C NMR (91 MHz, CD_3OD) δ 144.4 (C-5), 142.1 (C-6), 130.8 (C-3a), 130.7 (C-7a), 111.7 (C-4), 108.7 (C-7), 35.7 (C-2), 33.3 (C-3)

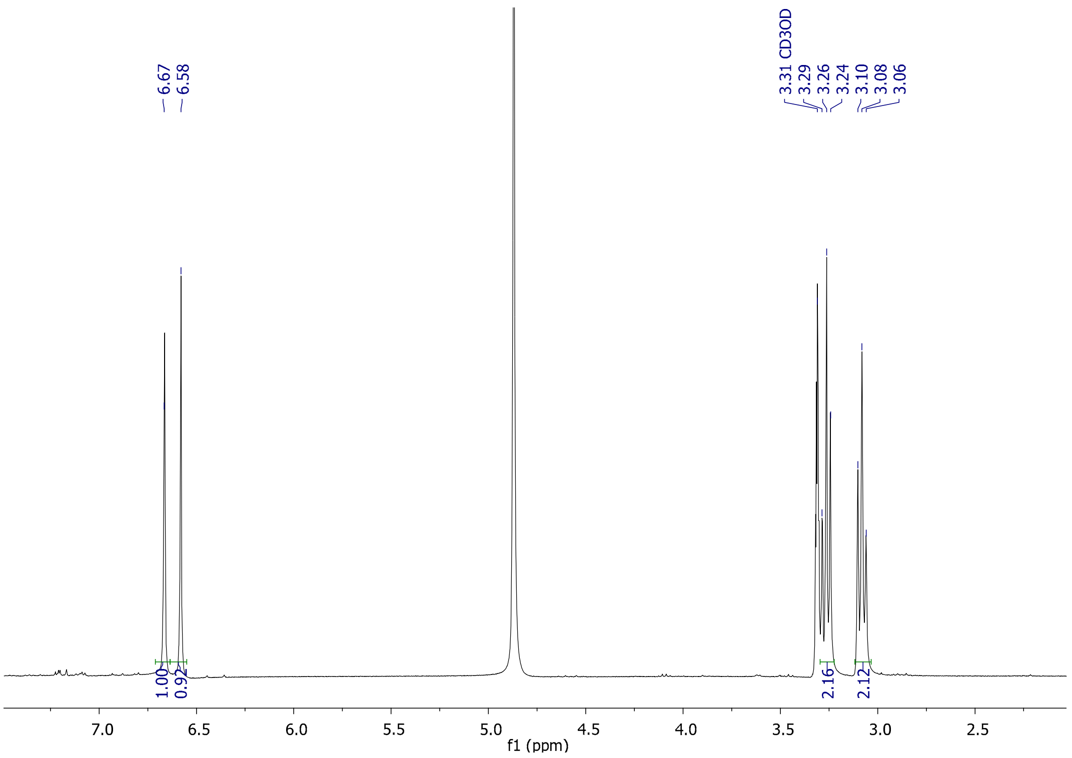

Figure S1. ^{1}H NMR spectrum of **7**

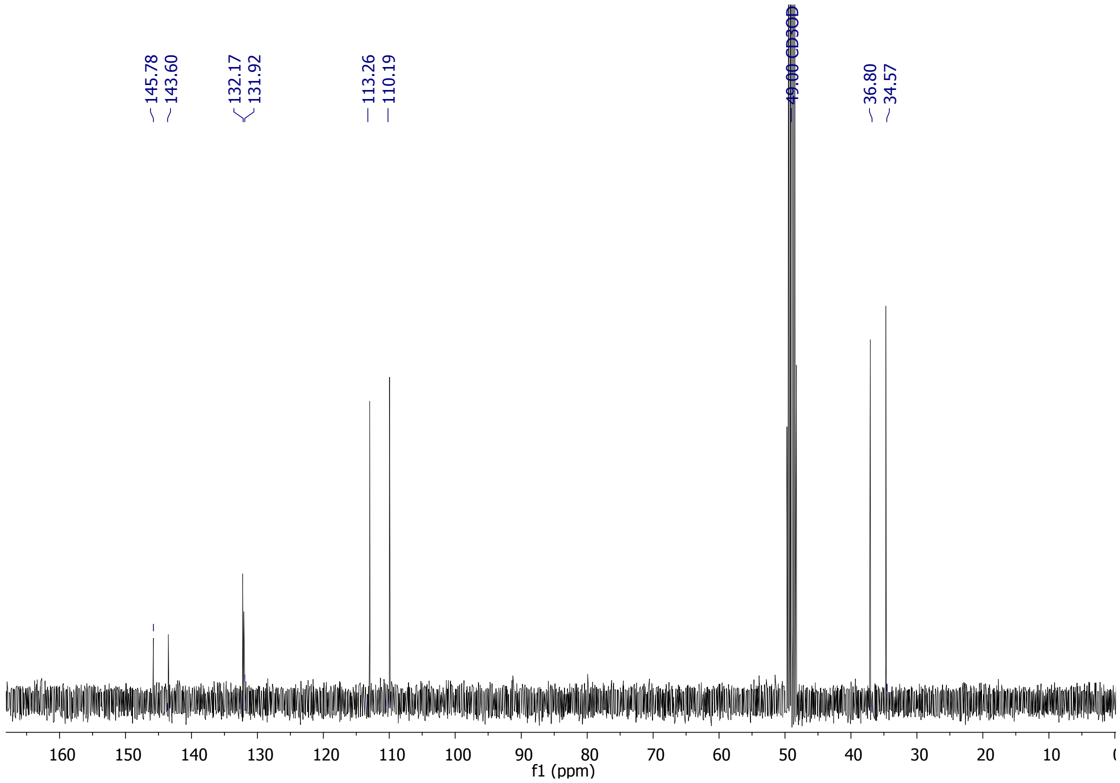

Figure S2. ^{13}C NMR spectrum of **7**

*Synthesis of 5,6-dihyroxy[b]benzothiophene (**8, DHBT**).*

H_2-DHBT (100 mg, 0.6 mmol) was dissolved in MeOH (1 mL) then 60 mL of the proper buffer were added: 0.05 M carbonate buffer pH 9 (O_2 mediated oxidation) or 0.05 M phosphate buffer pH 7.7 when the oxidation was performed under argon atmosphere by $K_3[Fe(CN)_6]$ (2 molar eq.). Suddenly, the reaction mixture turned deep red and slowly faded to almost colorless. After 24h or 30 min, the reaction was extracted with ethyl acetate, the organic layers dried over anidrous Na_2SO_4. DHBT (**8**) was recovered as a white solid without further purification (yield: 75% or 90%).

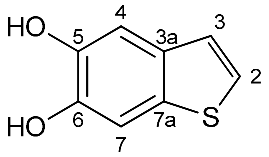

8

^{1}H NMR (400 MHz, MeOD) δ 7.21 (s, H-4), 7.22 – 7.20 (d, J = 5.4, H-2), 7.18 (s, H-7), 7.08 (dd, J = 5.4, 0.7 Hz, H-3); ^{13}C NMR (101 MHz, MeOD) δ 146.0 (C-6), 145.5 (C-5), 134.6 (C-3a), 132.9 (C7a), 124.3 (C-4), 124.0 (C-3), 109.1 (C-7), 108.0 (C-2).

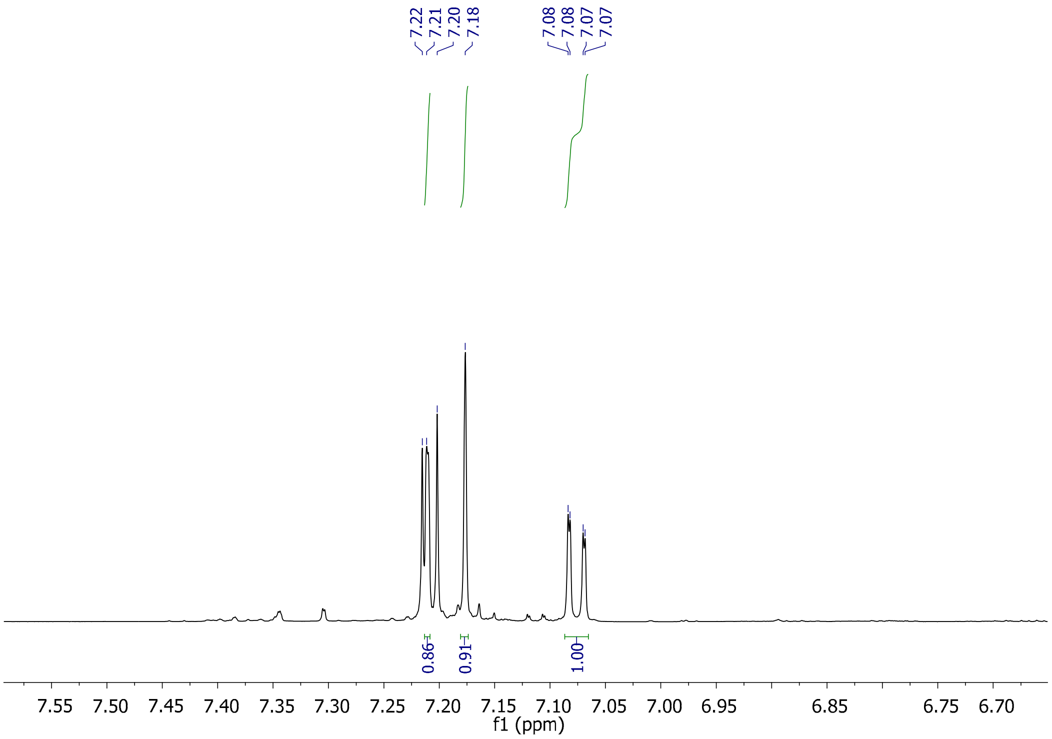

Figure S3. ^{1}H NMR spectrum of **8**

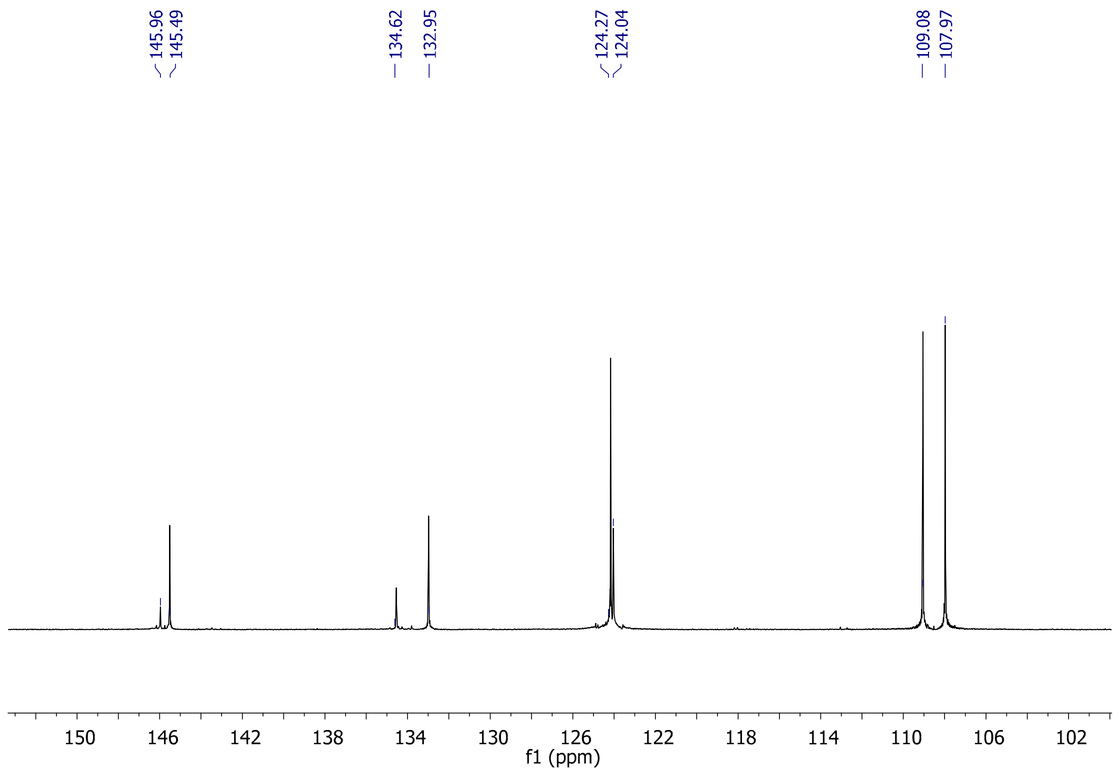

Figure S4. ^{13}C NMR spectrum of **8**

*Synthesis and characterization of 5,6-diacetoxybenzo[b]thiophene (**9**, **DABT**).* DHBT (100 mg, 0.60 mmol) was dissolved in acetic anhydride-pyridine (1 mL-20 L) and left under stirring overnight at room temperature. DABT was recovered as a white amorphous solid in very high yield (>> 99%) after solvent evaporation without further purification.

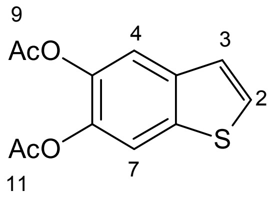

9

^{1}H NMR (500 MHz, $CDCl_3$) δ 7.69 (s, H-7), 7.62 (d, *J* = 1.1 Hz, H-4), 7.47 (dd, *J* = 5.2, 1.4 Hz, H-2), 7.30 – 7.27 (dd, *J* = 5.2, 1.4 Hz, H-3), 2.33 (s, H-9,11); ^{13}C NMR (126 MHz, $CDCl_3$) δ 168.8, 168.7 (C-8,10), 140.1, 139.8 (C-5,6), 137.8 (C-3a), 137.3 (C-7a), 128.3 (C-2), 123.6 (C-3), 117.4 (C-4), 116.7 (C-7), 20.8 (C-9,11)

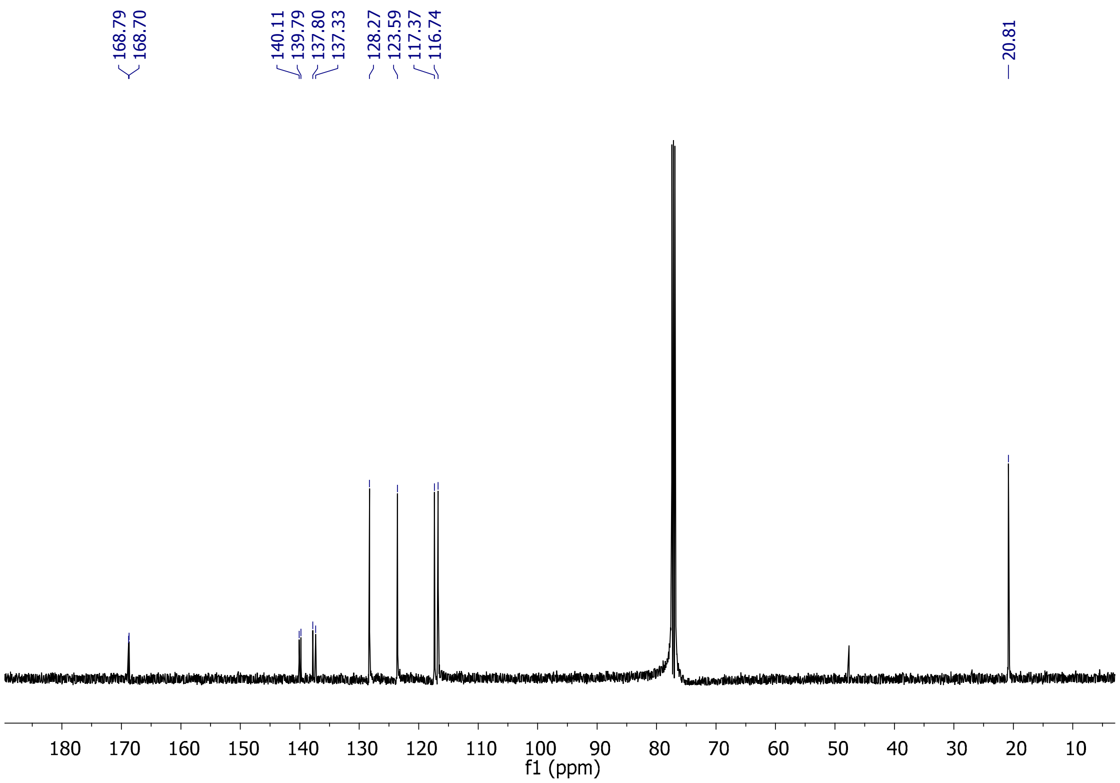

Figure S5. ^{13}C NMR spectrum of **9**

Oxidation of DHPET or DHBT. To a methanolic solution of DHPET or DHBT, the proper buffer was added to achieve the desired substrate concentration (1 or 10 mM). Different oxidative conditions were tested and used as required:

a) O_2 mediated oxidation in 0.05 M Na_2CO_3 buffer pH 8.5-9;

b) sodium periodate (1-2 molar eq.) in 0.1 M phosphate buffer (pH 7.4);

c) Cerium Ammonium Nitrate (1-2 molar eq.) in 0.1 M phosphate buffer (pH 3.0);

d) potassium ferricyanide (1-3 molar eq.) in 0.05 M Na_2CO_3 buffer pH 7.7;

e) Horse radish peroxidise (HRP)/H_2O_2 (50 U mL^{-1}, 1 molar eq.) in 0.05 M phosphate buffer pH 7.7.

Substrate consumption was determined by TLC ($CHCl_3$:AcOEt 8:2 or hexane:AcOEt 7:3) or HPLC analysis (0.1 % HCOOH-ACN 8:2).

Synthesis and structural characterization of DHPET dimers. Dimers were obtained oxidizing DHPET (50 mg, 0.29 mmol) in the conditions described at line e) of the previous paragraph (30 mL buffer). Purification was achieved by Preparative Layer Chromatography (PLC), $CHCl_3$:AcOEt 8:2 was selected as eluant. Two fractions were collected, referred as **2** and **3,** corresponding respectively to the thiosulfinate (yield: 22%) and the disulfide (yield: 35%) dimeric species.

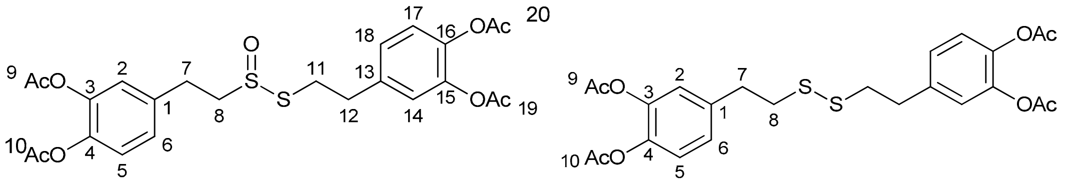

Figure S6. Hypothetical structures of the main oxidation products from DHPET: Fraction 1 (left), fraction 2 (right)

2: R_f: 0.8 ($CHCl_3$:AcOEt 8:2), ESI$^+$-TOF LC-MS: *m/z* 545 $[M+Na]^+$, ^1H NMR (400 MHz, $CDCl_3$), ^{13}C NMR (101 MHz, $CDCl_3$) δ 168.4 (C-9), 142.1 (C-3), 140.9 (C-4), 137.9 (C-1), 137.0 (C-6), 126.9 (C-2), 123.7 (C-5), 56.5 (C-8), 39.4 (C-10), 36.5 (C-7), 33.9 (C-11), 20.6 (C-9).

3: R_f: 0.6 ($CHCl_3$:AcOEt 8:2), ESI$^+$-TOF LC-MS: *m/z* 529 $[M+Na]^+$, ^1H NMR (400 MHz, $CDCl_3$) δ 7.10 (s, H-2), 7.09 (d, H-5), 7.03 (d, H-6), 3.01 – 2.94 (t, J= 2.98 Hz, H-7), 2.93 – 2.86

(t, J= 2.90 Hz, H-8), 2.28 (s, H-9); ^{13}C NMR (101 MHz, $CDCl_3$) δ 168.4 (C-9,10), 141.9 (C-3), 140.4 (C-4), 138.8 (C-1), 126.8 (C-6), 123.5,(C-2) 123.3 (C-5), 39.4 (C-8), 34.9 (C-7).

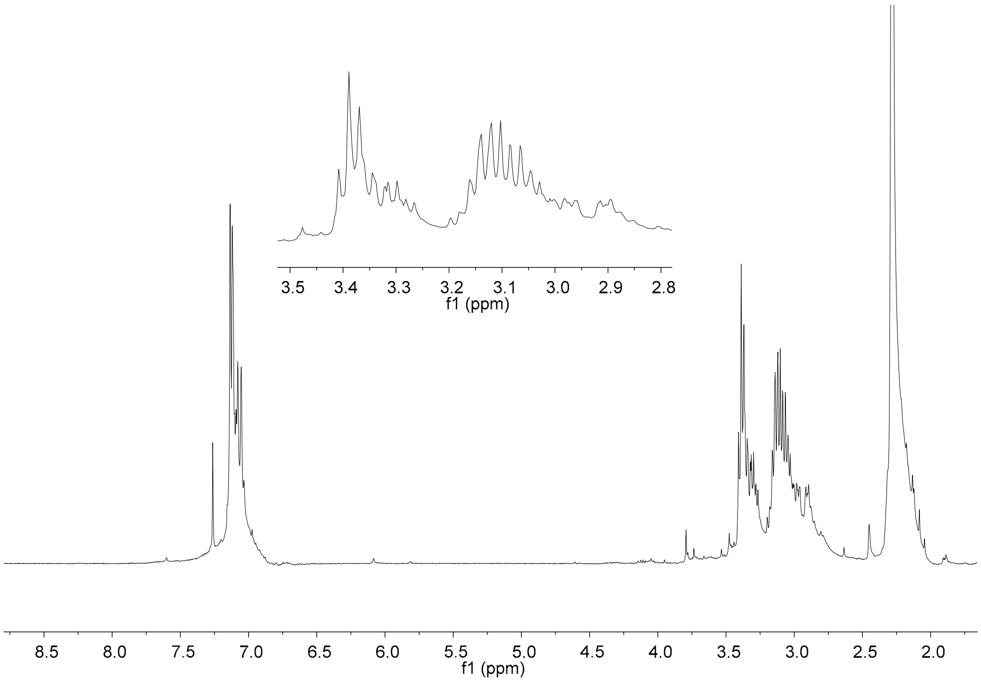

Figure S7. ^{1}H NMR spectrum of **2**

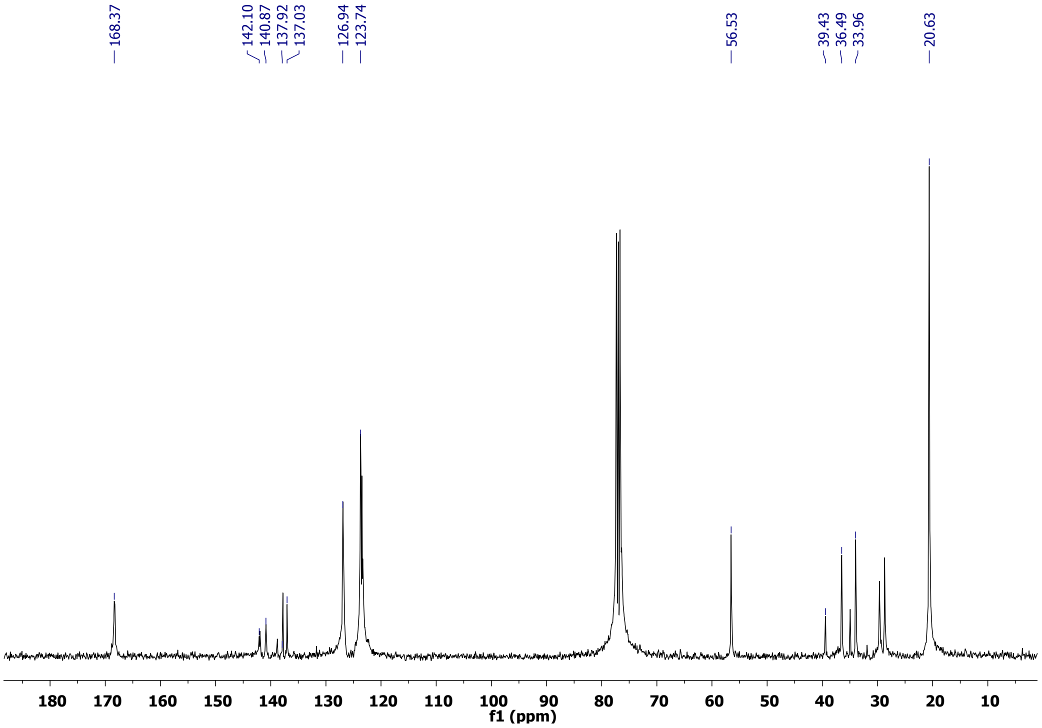

Figure S8. ^{13}C NMR spectrum of **2**

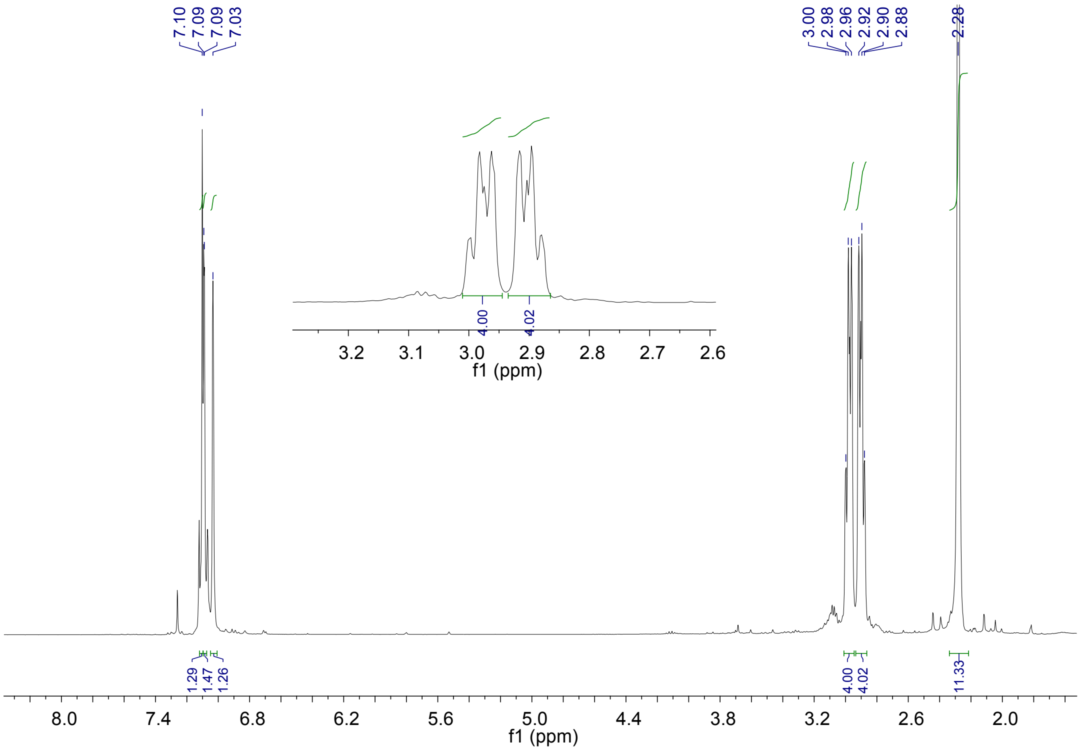

Figure S9. ^{1}H NMR spectrum of **3**

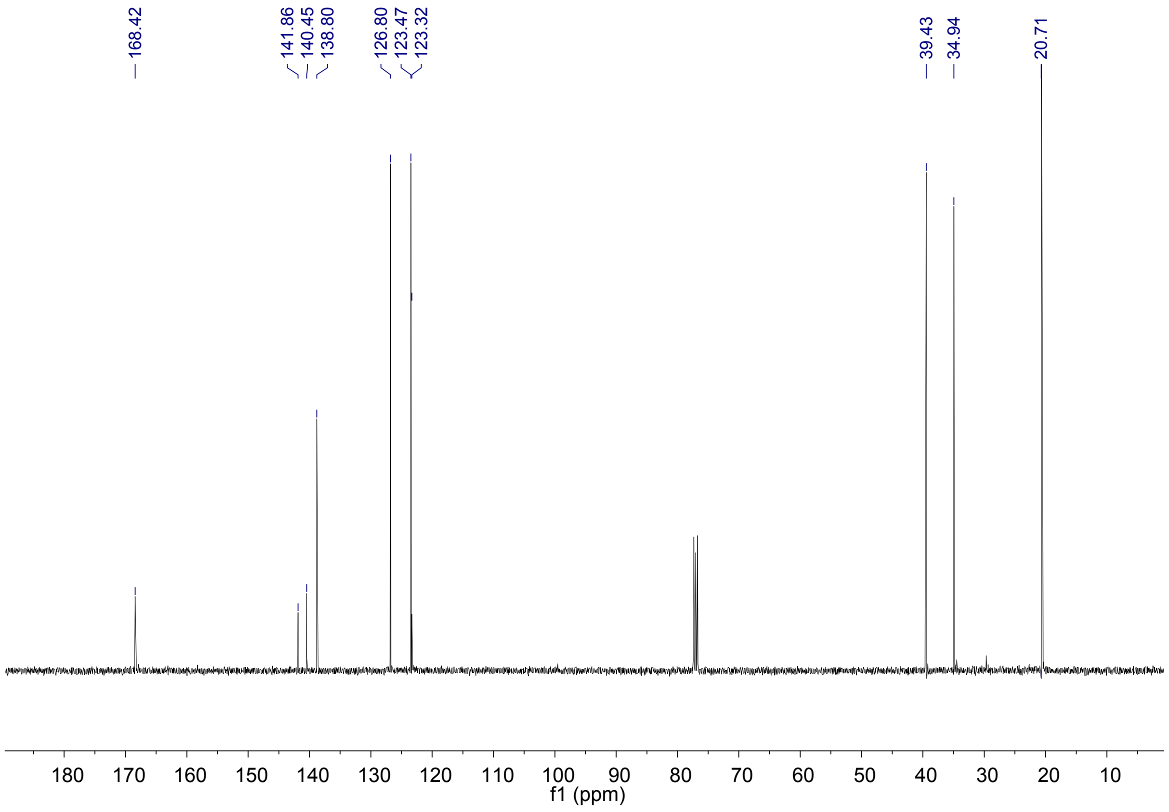

Figure S10. ^{13}C NMR spectrum of **3**

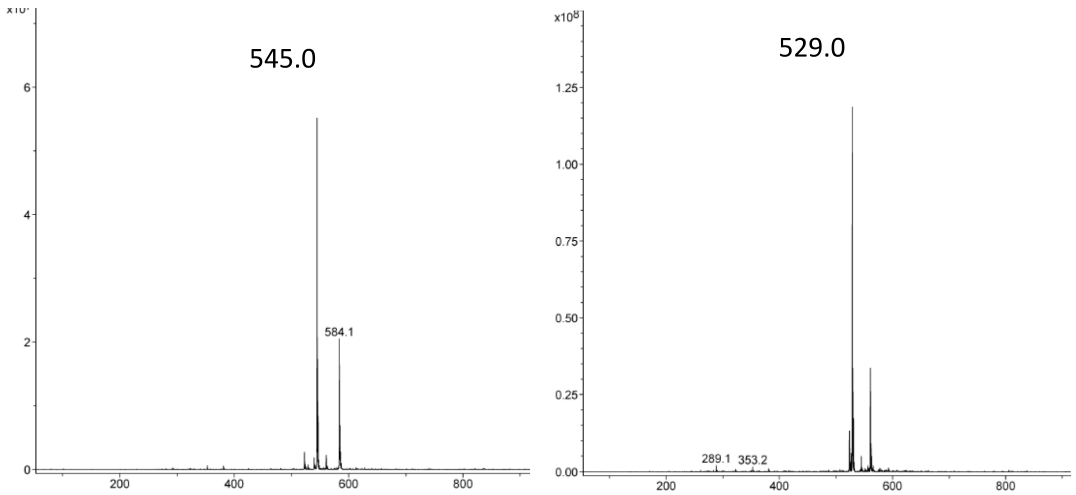

Figure S11. MS-analysis of fraction 1 (compound **2**), left; and fraction 2 (compound **3**), right

***Synthesis and preliminar structural characterization of acetylated DHBT oligomers**.* For analytical purposes, a 10 mM stock solution in MeOH of DHBT was diluted 10 times in the proper buffer, then the proper oxidant was added (condition described in lines from a) to e)). Reaction courses were monitored by HPLC. In the casa of line c) to e), the mixtures were extracted (water/ethyl acetate) organic layer acetylated and analysed by ESI^+ LC-MS spectrometry (Figure S12). For preparative purposes DHBT (50 mg, 0.30 mmol) was dissolved in methanol (1 mL) oxidation with 2 molar eq of potassium ferrycianide in 50 mM carbonate buffer pH 8 (30 mL). The reaction was left under stirring, in argon atmosphere, at room temperature for 45 min. The reaction mixture was then reduced by sodium diotionite and extracted in ethyl acetate. The organic layers (c.a. 30 mg, 60% w/w of starting material) were dried over anhydrous Na_2SO_4, evaporated and subsequently acetylated by acetic anhydride- pyridine (1 mL- 20 μL) overnight. Purification of the acetylated oligomers mixture was performed by silica gel chromatography whit hexane:ethyl acetate 7:3 as the eluant. Besides a first fraction mainly consisting of the acetylated monomer (DABT, R_f: 0.6, yield: 25%) other three fractions were collected referred to as Fraction I to III (R_f: 0.4, 0.2 and 0.1, respective yields: 4%, 9% and 2%).

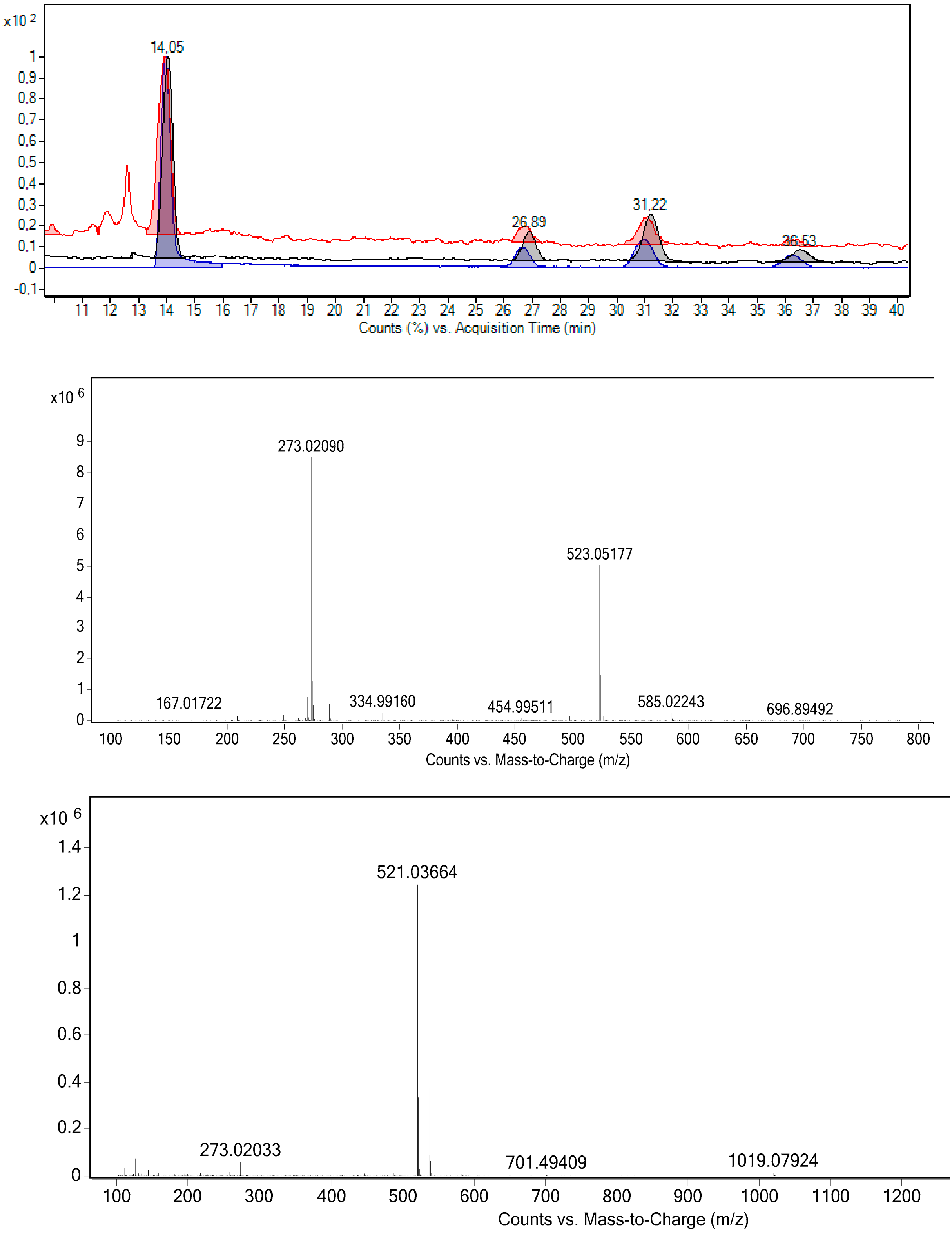

Figure S12. Top) TIC traces of the acetylated organic extracts from DHBT oxidation with (HRP)/H_2O_2 (50 U mL^{-1}, 1 eq, red), $K_3[Fe(CN)_6]$ (1 eq, black) or $(NH_4)Ce(NO_3)_6$(1 eq, blue). Middle) ESI$^+$ LC-MS spectrum of the peak with R_t= 14 min corresponding to DABT. Bottom) ESI$^+$ LC-MS spectrum of the peak with R_t= 27, 31 or 36 min corresponding to three isomeric dimers with the same m/z = 521.

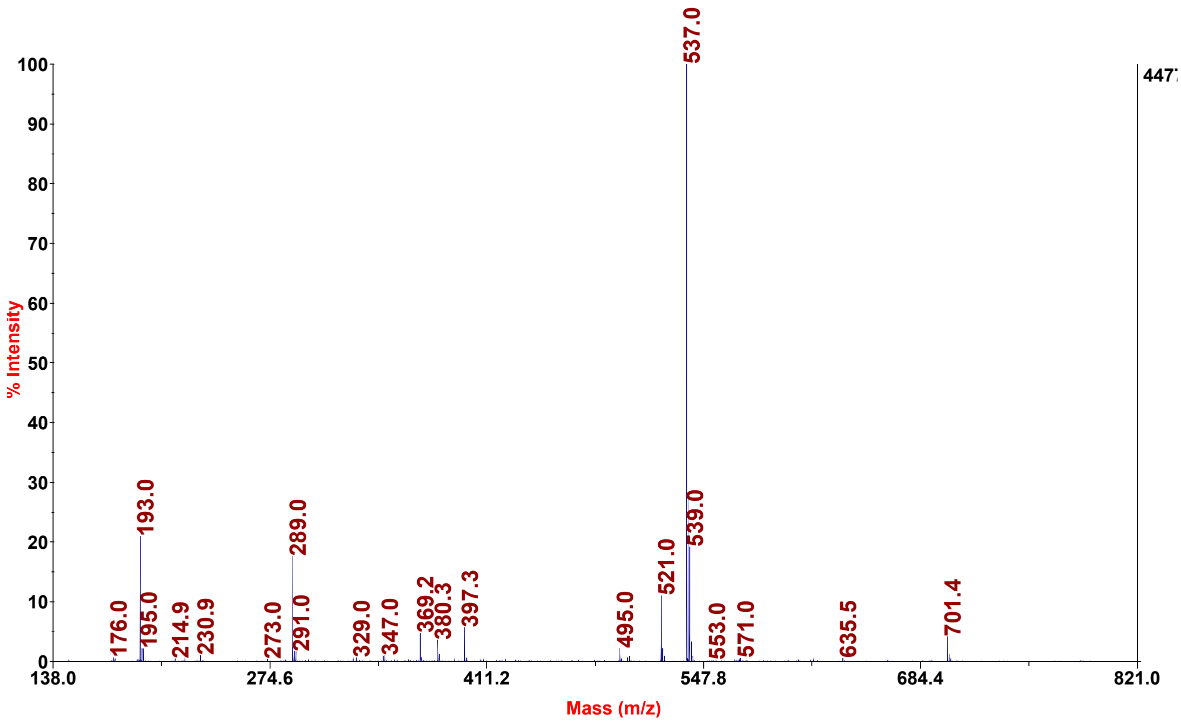

Figure S13. MALDI-MS spectrum of fraction I (mixture of acetylated dimers of DHBT).

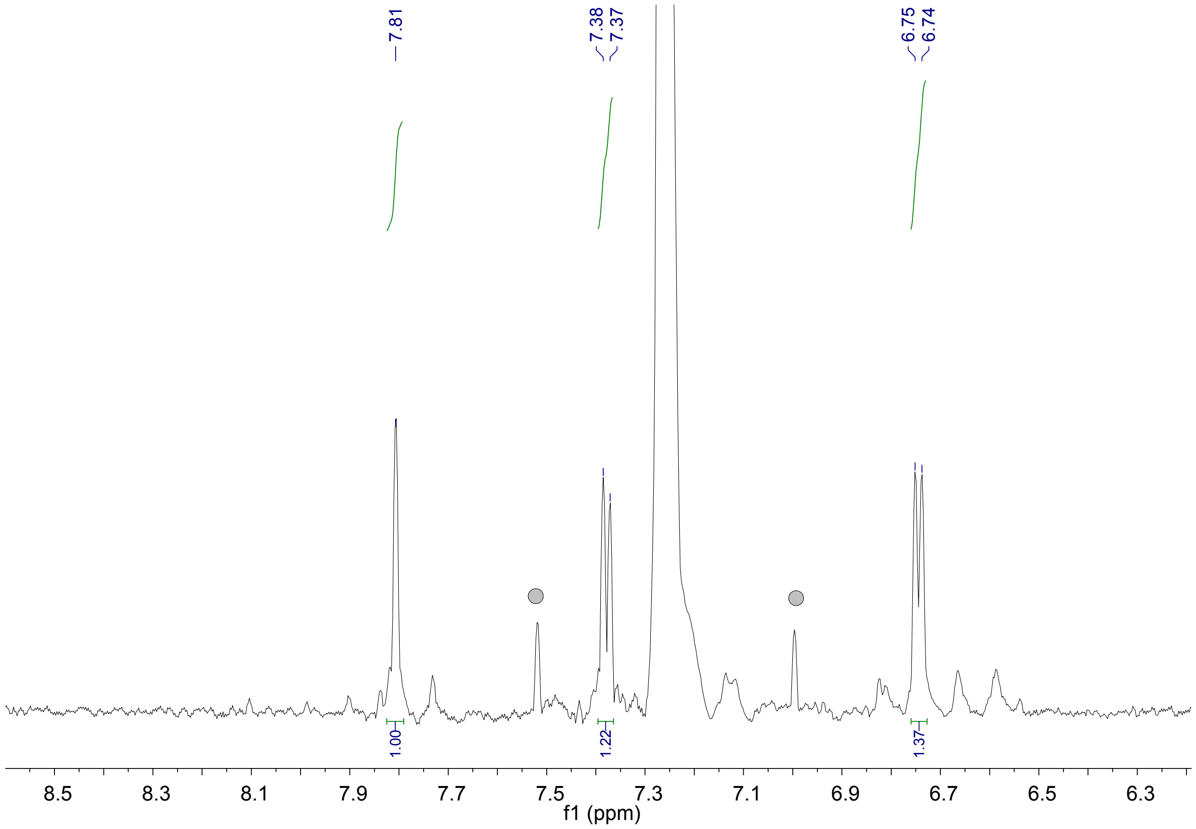

Figure S14. Expansion (8.5-6.0 ppm) of the ^{1}H NMR spectrum in $CDCl_3$ of fraction I. Solvent side bands are marked by a dot.

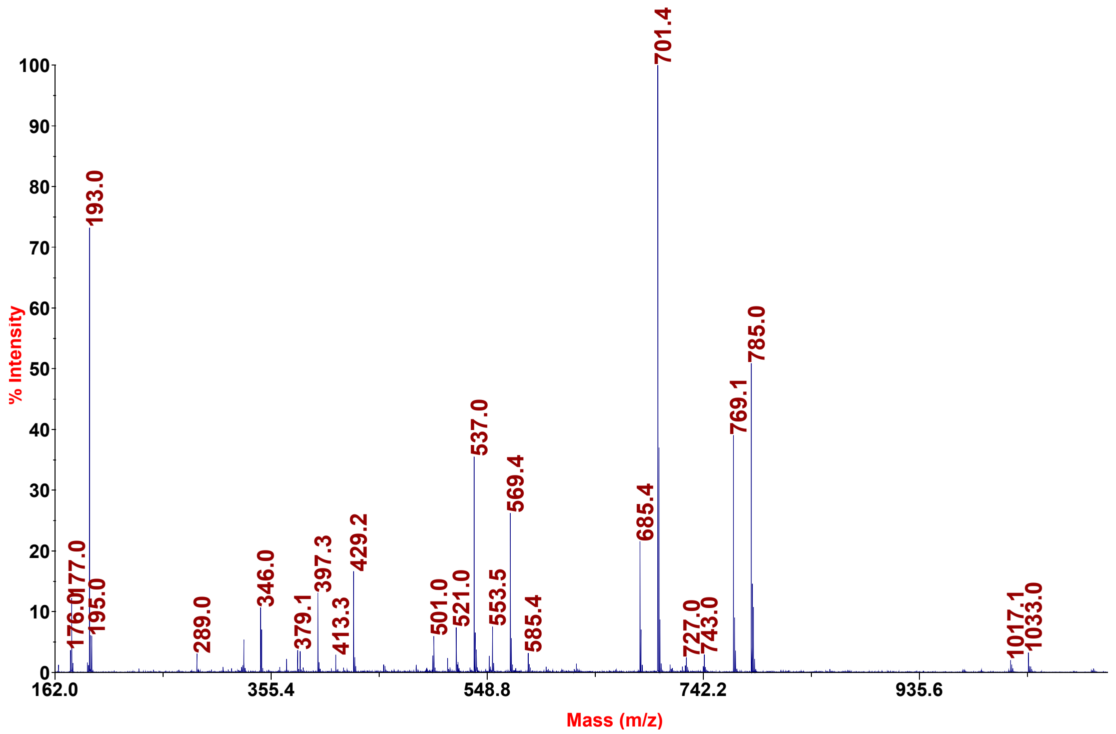

Figure S15. MALDI-MS spectrum of fraction II (mixture of acetylated trimers of DHBT).

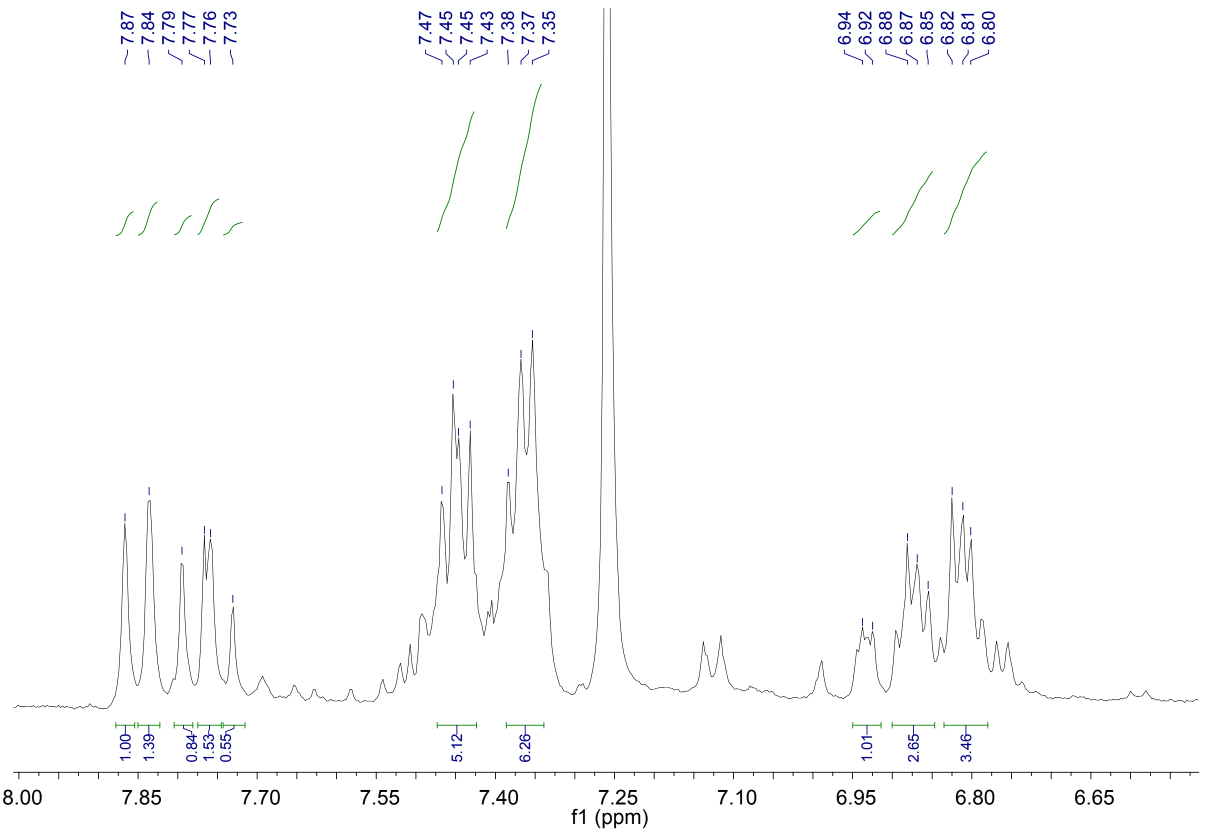

Figure S16. Expansion (8.0-6.5 ppm) of the ^1H NMR spectrum in $CDCl_3$ of fraction II.

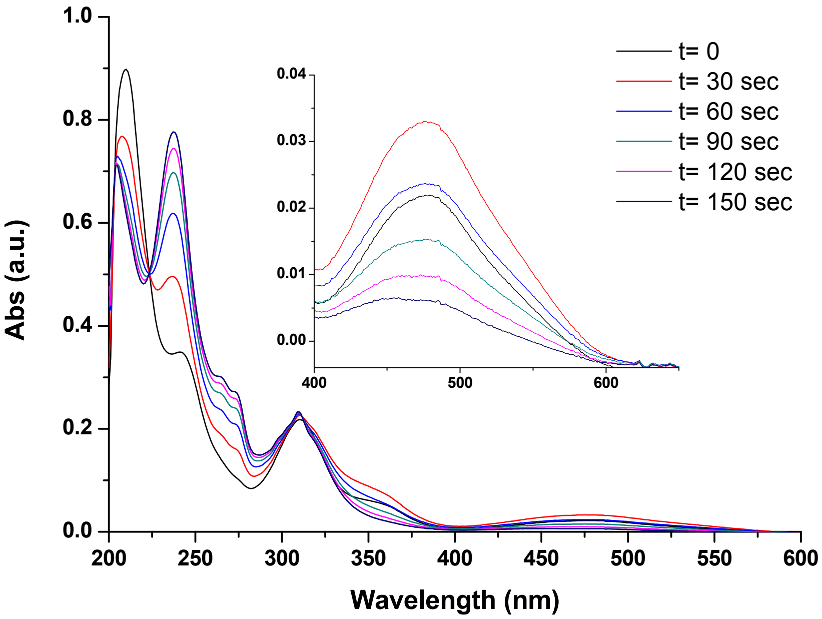

Figure S17. Spectrophotometric analysis of 0.05 mM H_2-DHBT autoxidation in 50 mM pH 8.8 phosphate buffer.

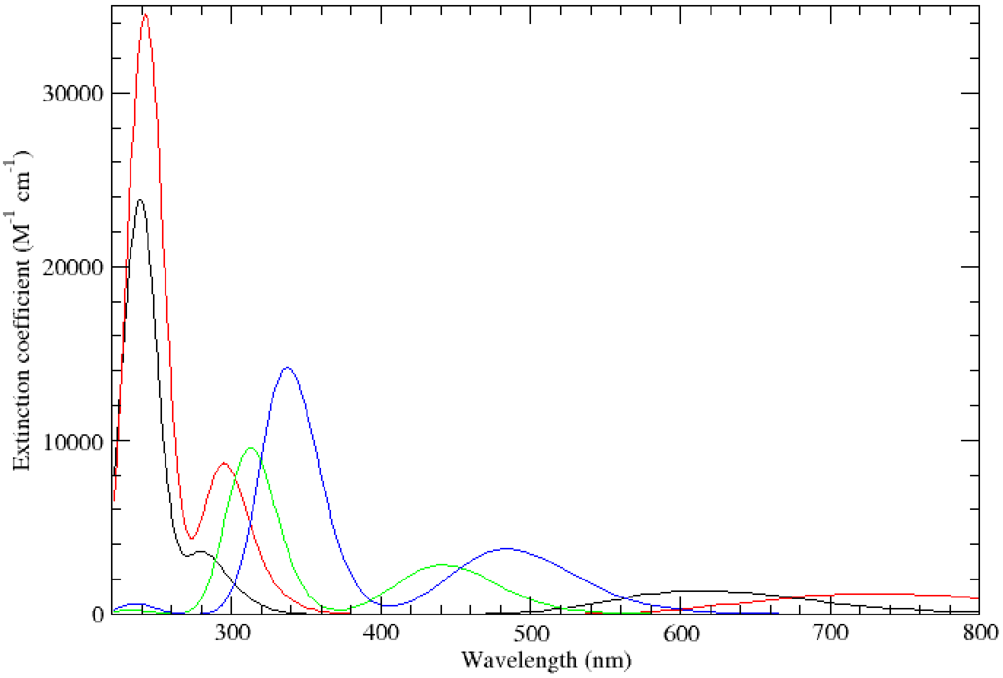

Figure S18. Spectra of *o*-quinones from DHBT and from H_2-DHBT, computed at the TD-PBE0/6-311++G(2d,2p)/PCM level both in vacuo and in water (PCM). Black line, DHBT *o*-quinone in vacuo; red line, DHBT *o*-quinone in water; green line, H_2-DHBT *o*-quinone in vacuo; blue line, H_2-DHBT *o*-quinone in water.

Table S1. Comparison between experimental NMR chemical shifts (ppm) of DHBT and DHI and their respective peracetylated derivatives (DABT, DAI).

	**DHBT/DABT**		**DHI/DAI**	
**Position**	**(^{1}H)**	**(^{13}C)**	**(^{1}H)**	**(^{13}C)**
2	7.21/7.48	108.0/128.3	6.20/6.45	122.8/127.5
3	7.08/7.28	124.0/123.6	6.98/7.41	101.3/101.6
3a		134.6/137.8		142.3/137.8
4	7.21/7.59	124.3/117.4	6.89/7.36	104.9/113.7
5		145.5/139.8		130.6/133.5
6		146.0/140.1		140.5/136.3
7	7.18/7.69	109.1/116.7	6.82/7.27	98.7/106.1
7a		132.9/137.3		120.9/125.6
AcetylC=O		168.8 and 168.9		169.5
Me	2.33	20.8		21.0

Table S2. NMR chemical shifts of peracetylated DHBT oligomers, computed at the PBE0 / 6-311+G(d,p) // PBE0-D3BJ / 6-31+G(d,p) level in $CDCl_3$.

**Ring**	**Position**	**Monomer**[a]	**4,4′-dimer**[b,c]	**7,7′-dimer**[d,e]	**4,7′-dimer**[f]	**4,4′:7′,7″-trimer**[g]
			**H, ppm**			
1	H2	7.268	7.189	7.328	7.206	7.270
1	H3	7.208	6.698	7.239	6.722	6.640
1	H4	7.497	-	7.609	-	-
1	H7	7.518	7.656	-	7.627	7.657
1	H(Me)-Ac6	1.839	1.661	1.802	1.658	1.727
1	H(Me)-Ac7	1.841	1.900	1.504	1.904	1.914
2	H2				7.331	7.255
2	H3				7.218	6.684
2	H4				7.562	-
2	H7				-	-
2	H(Me)-Ac6				1.913	1.675
2	H(Me)-Ac7				1.671	1.726
3	H2					7.431
3	H3					7.255
3	H4					7.646
3	H7					-
3	H(Me)-					1.913

	Ac6					
3	H(Me)-Ac7					1.684
			**c, ppm**			
1	C2	132.651	132.788	133.469	132.612	133.030
1	C3	122.733	123.558	122.829	123.785	123.431
1	C4	116.958	124.864	118.268	124.229	124.552
1	C5	140.482	138.724	141.136	138.037	138.919
1	C6	140.174	141.555	137.947	140.768	140.949
1	C7	116.450	116.713	123.464	117.265	116.703
1	C3a	136.458	136.734	135.870	135.586	136.786
1	C7a	139.753	140.358	141.207	140.765	140.116
1	C(=O)-Ac6	171.832	172.552	171.885	172.358	172.294
1	C(Me)-Ac6	16.532	16.293	16.716	16.128	16.688
1	C(=O)-Ac7	171.798	173.069	172.721	173.066	172.852
1	C(Me)-Ac7	16.458	16.570	16.120	16.509	16.544
2	C2				133.635	134.115
2	C3				122.515	122.901
2	C4				117.155	125.282
2	C5				142.016	139.177
2	C6				138.636	138.306
2	C7				124.831	124.119
2	C3a				136.698	136.681
2	C7a				141.814	140.379
2	C(=O)-Ac6				173.035	173.010
2	C(Me)-Ac6				16.496	16.363
2	C(=O)-Ac7				172.499	172.934
2	C(Me)-Ac7				16.424	16.334
3	C2					133.975
3	C3					122.641
3	C4					117.594
3	C5					141.468
3	C6					138.405
3	C7					123.895
3	C3a					136.838
3	C7a					140.417
3	C(=O)-Ac6					172.932
3	C(Me)-Ac6					16.556
3	C(=O)-Ac7					172.103
3	C(Me)-Ac7					16.518

[a] 192 conformations were explored by a rigid scan at the PM6 level. 32 conformers were fully optimized at the PBE0-D3BJ / 6-31+G(d,p) level in $CDCl_3$. The reported chemical shifts are Boltzmann averages over the 7 most significant conformers.
[b] 512 conformations were explored by a rigid scan at the PM6 level. 26 conformerswere fully optimized at the PBE0-D3BJ / 6-31+G(d,p) level in $CDCl_3$. The reported chemical shifts are Boltzmann averages over the 6 most significant conformers.
[c] Shifts averaged over the two rings.
[d] 512 conformations were explored by a rigid scan at the PM6 level. 14 conformerswere fully optimized at the PBE0-D3BJ / 6-31+G(d,p) level in $CDCl_3$. The reported chemical shifts are Boltzmann averages over the 11 most significant conformers.
[e] Shifts averaged over the two rings.
[f] 512 conformations were explored by a rigid scan at the PM6 level. 27 conformerswere fully optimized at the PBE0-D3BJ / 6-31+G(d,p) level in $CDCl_3$. The reported chemical shifts are Boltzmann averages over the 10 most significant conformers.
[gconformations were explored by a rigid scan at the PM6 level. 134 conformerswere fully optimized at the PBE0-D3BJ / 6-31+G(d,p) level in $CDCl_3$. The reported chemical shifts are Boltzmann averages over the 46 most significant conformers.

Table S3. Mulliken spin densities (with hydrogens summed into heavy atoms) computed in water (PBE0 / 6-31+G(d,p) / SMD) for DHBT and for DHI semiquinones.

Position	DHBT Semiquinone			DHI Semiquinone		
	Neutral form, 5-yl radical	Neutral form, 6-yl radical	Anionic form	Neutral form, 5-yl radical	Neutral form, 6-yl radical	Anionic form
**C2**	0.012	0.282	0.157	0.055	0.304	0.201
**C3**	0.027	-0.133	-0.078	-0.003	-0.116	-0.118
**C4**	0.229	-0.216	-0.028	0.215	-0.165	-0.012
**C7**	-0.155	0.308	0.090	-0.150	0.206	0.003

Table S4. Relative stabilities of different positional isomers of the first-formed dimeric products arising from coupling of two DHBT semiquinones, one of them being in anionic form.

Dimer	Coupling mode	$\Delta G_{SMD,RRHO}$ (kcal mol^{-1})
**2,2′**	2 of 6-yl semiquinone on 2 of semiquinone anion	0.15
**2,4′**	4 of 5-yl semiquinone on 2 of semiquinone anion	1.23
	2 of 6-yl semiquinone on 4 of semiquinone anion	0.40
**2,7′**	2 of 6-yl semiquinone on 7 of semiquinone anion	2.08
	7 of 6-yl semiquinone on 2 of semiquinone anion	1.47
**4,4′**	4 of 5-yl semiquinone on 4 of semiquinone anion	**0.00**
**4,7′**	4 of 5-yl semiquinone on 7 of semiquinone anion	2.14
	7 of 6-yl semiquinone on 4 of semiquinone anion	0.43
**7,7′**	7 of 6-yl semiquinone on 7 of semiquinone anion	3.04

Table S5. Conformational exploration of the first-formed dimeric products arising from coupling of two neutral DHBT semiquinones.

Dimer	Coupling mode	Diastereoisomer	Symmetry point group[a]	Interring dihedral (deg)[b]	$G_{el,PCM}$ (Ha)[c]	$H_{PCM,RRHO}$ (Ha)[d]	$G_{PCM,RRHO}$ (Ha)[e]	$G_{el,SMD}$ (Ha)[f]	$G_{SMD,RRHO}$ (Ha)[g]	$G_{SMD,RRHO}$ (kcal mol^{-1})[h]
**2,2′**	2 of 6-yl semiquinone on 2 of 6-yl semiquinone	*dl*	C_2	-57.1	-1711.757425	-1711.511786	-1711.577599	-1711.767020	-1711.587194	2.99
			C_2	64.0	-1711.760686	-1711.515136	-1711.580688	-1711.770377	-1711.590379	0.99
			C_2	-171.9	-1711.759030	-1711.513419	-1711.578956	-1711.767601	-1711.587527	2.78
		meso		-61.5	-1711.760027	-1711.514370	-1711.580479	-1711.769484	-1711.589936	1.27

			C_i	180.0	-1711.757727	-1711.512074	-1711.578469	-1711.766312	-1711.587054	3.08
**2,4′**	2 of 6-yl semiquinone on 4 of 5-yl semiquinone	*RR*		-70.0	-1711.761696	-1711.516023	-1711.582203	-1711.769730	-1711.590237	1.08
				68.5	-1711.759013	-1711.513294	-1711.579081	-1711.767018	-1711.587086	3.06
				178.9	-1711.756041	-1711.510254	-1711.576273	-1711.763748	-1711.583980	5.00
		RS		-65.7	-1711.755747	-1711.509944	-1711.576454	-1711.763741	-1711.584448	4.71
				65.6	-1711.761399	-1711.515793	-1711.581629	-1711.769949	-1711.590179	1.12
				-162.0	-1711.760491	-1711.514778	-1711.580817	-1711.768452	-1711.588778	1.99
**2,7′**	2 of 6-yl semiquinone on 7 of 6-yl semiquinone	*RR*		-63.1	-1711.754168	-1711.508338	-1711.574565	-1711.761886	-1711.582283	6.07
				55.7	-1711.760855	-1711.515265	-1711.581507	-1711.768688	-1711.589340	1.64
				-169.4	-1711.757262	-1711.511503	-1711.577205	-1711.764788	-1711.584731	4.53
		RS		-59.2	-1711.759628	-1711.513983	-1711.579935	-1711.767774	-1711.588081	2.43
				73.1	-1711.760121	-1711.514395	-1711.580377	-1711.768151	-1711.588407	2.23
				170.4	-1711.753371	-1711.507627	-1711.574309	-1711.760808	-1711.581746	6.41
**4,4′**	4 of 5-yl semiquinone on 4 of 5-yl semiquinone	*dl*	C_2	-48.6	-1711.758666	-1711.513233	-1711.578600	-1711.766789	-1711.586723	3.28
			C_2	-61.0	-1711.760137	-1711.514373	-1711.578181	-1711.768141	-1711.586185	3.62
			C_2	55.2	-1711.752836	-1711.506965	-1711.571447	-1711.760155	-1711.578766	8.28
			C_2	144.5	-1711.763630	-1711.518215	-1711.583736	-1711.771060	-1711.591166	0.50
		meso		-72.9	-1711.763833	-1711.518295	-1711.584882	-1711.770907	-1711.591956	0.00
			C_i	180.0	-1711.751395	-1711.505437	-1711.570450	-1711.759775	-1711.578830	8.24
			C_i	180.0	-1711.724124	-1711.478835	-1711.544381	-1711.731812	-1711.552069	25.03
**4,7′**	4 of 5-yl semiquinone on 7 of 6-yl semiquinone	*RR*		-57.8	-1711.763533	-1711.517944	-1711.583487	-1711.770518	-1711.590472	0.93
				57.5	-1711.749126	-1711.503338	-1711.568657	-1711.756854	-1711.576385	9.77
				159.8	-1711.762284	-1711.516650	-1711.582714	-1711.768427	-1711.588857	1.94
		RS		-82.7	-1711.762354	-1711.516892	-1711.583125	-1711.769115	-1711.589886	1.30
				80.9	-1711.757339	-1711.511810	-1711.577750	-1711.765092	-1711.585503	4.05
				61.5	-1711.758868	-1711.513127	-1711.577599	-1711.766462	-1711.585193	4.24
				174.8	-1711.721840	-1711.476769	-1711.542852	-1711.729006	-1711.550018	26.32
				175.6	-1711.750190	-1711.504445	-1711.570461	-1711.757315	-1711.577586	9.02
**7,7′**	7 of 6-yl semiquinone on 7 of 6-yl semiquinone	*dl*	C_2	-49.8	-1711.761005	-1711.515523	-1711.581153	-1711.767112	-1711.587260	2.95
			C_2	47.2	-1711.747687	-1711.501791	-1711.566535	-1711.755185	-1711.574033	11.25
			C_2	175.3	-1711.757455	-1711.511636	-1711.575550	-1711.764512	-1711.582607	5.87
			C_2	149.0	-1711.755630	-1711.510181	-1711.575462	-1711.762502	-1711.582334	6.04
		meso		-70.1	-1711.761942	-1711.516269	-1711.581675	-1711.768192	-1711.587925	2.53
			C_i	180.0	-1711.746727	-1711.501021	-1711.567503	-1711.753420	-1711.574196	11.14
			C_i	180.0	-1711.721743	saddle point		-1711.728213		

[a] C_1 if not specified.

[b] Fiducial groups are the highest priority atoms on either side of the interring C-C bond.

[c] Electronic energy in water (including electrostatic PCM contributions).

[d] Sum of electronic and thermal enthalpies in water (including electrostatic PCM contributions).

[e] Sum of electronic and thermal free energies in water (including electrostatic PCM contributions).

[f] Electronic energy in water (including both electrostatic and non-electrostatic SMD contributions).

[g] $G_{SMD,RRHO} = G_{PCM,RRHO} - G_{el,PCM} + G_{el,SMD}$.

[h] Relative $G_{SMD,RRHO}$ values referred to the most stable structure identified in the series.

Table S6. Conformational exploration of the first-formed dimeric products arising from coupling of two DHBT semiquinones, one of them being in anionic form.

Dimer	Coupling mode	Diastereoisomer	Interring dihedral (deg)[a]	$G_{el,PCM}$ (Ha)[b]	$H_{PCM,RRHO}$ (Ha)[c]	$G_{PCM,RRHO}$ (Ha)[d]	$G_{el,SMD}$ (Ha)[e]	$G_{SMD,RRHO}$ (Ha)[f]	$G_{SMD,RRHO}$ (kcal mol^{-1})[g]
**2,2′**	2 of 6-yl semiquinone on 2 of semiquinone anion	*RR*	-57.8	-1711.280641	-1711.048675	-1711.115253	-1711.295069	-1711.129681	2.19
			64.0	-1711.283421	-1711.051592	-1711.118165	-1711.298181	-1711.132925	0.15
			-172.0	-1711.281300	-1711.049498	-1711.115696	-1711.295451	-1711.129847	2.09
		RS	-60.4	-1711.282366	-1711.050524	-1711.116560	-1711.297105	-1711.131299	1.18
			63.5	-1711.282653	-1711.050809	-1711.117569	-1711.297148	-1711.132064	0.70
			-179.8	-1711.280594	-1711.048810	-1711.115424	-1711.294481	-1711.129311	2.42
**2,4′**	4 of 5-yl semiquinone on 2 of semiquinone anion	*RR*	-69.8	-1711.283913	-1711.052034	-1711.118101	-1711.297022	-1711.131210	1.23
			69.0	-1711.280387	-1711.048386	-1711.113932	-1711.293949	-1711.127494	3.56
			176.5	-1711.277857	-1711.045863	-1711.112430	-1711.291173	-1711.125746	4.66
		RS	-66.2	-1711.277346	-1711.045361	-1711.111742	-1711.290836	-1711.125232	4.98
			63.6	-1711.282247	-1711.050442	-1711.116685	-1711.296696	-1711.131134	1.28
			-162.7	-1711.282126	-1711.050193	-1711.116535	-1711.295653	-1711.130062	1.95
	2 of 6-yl semiquinone on 4 of semiquinone anion	*RR*	-70.2	-1711.285856	-1711.054008	-1711.119935	-1711.298405	-1711.132484	0.43
			67.5	-1711.285268	-1711.053430	-1711.118805	-1711.296531	-1711.130068	1.95
			-178.6	-1711.281861	-1711.049966	-1711.116301	-1711.293410	-1711.127850	3.34
		RS	-59.2	-1711.281658	-1711.049841	-1711.116155	-1711.293093	-1711.127590	3.50
			67.4	-1711.285552	-1711.053811	-1711.119599	-1711.298486	-1711.132533	0.40
			-160.9	-1711.284864	-1711.052958	-1711.118677	-1711.297147	-1711.130960	1.39
**2,7′**	2 of 6-yl semiquinone on 7 of semiquinone anion	*RR*	-61.2	-1711.277483	-1711.045458	-1711.111641	-1711.289790	-1711.123948	5.79
			56.4	-1711.282640	-1711.050826	-1711.116856	-1711.295639	-1711.129855	2.08
			-170.8	-1711.281480	-1711.049511	-1711.114846	-1711.292991	-1711.126357	4.28
		RS	-59.1	-1711.281594	-1711.049717	-1711.115347	-1711.294976	-1711.128729	2.79
			74.1	-1711.281959	-1711.050025	-1711.116437	-1711.295126	-1711.129604	2.24
			74.3	-1711.281982	-1711.050056	-1711.116009	-1711.295085	-1711.129112	2.55
			-179.3	-1711.277322	-1711.045371	-1711.110984	-1711.288900	-1711.122562	6.66
			162.6	-1711.275551	-1711.043725	-1711.109169	-1711.287282	-1711.120900	7.70
	7 of 6-yl semiquinone on 2 of semiquinone anion	*RR*	-66.9	-1711.276233	-1711.044211	-1711.110155	-1711.289456	-1711.123378	6.15
			-64.5	-1711.276204	saddle point		-1711.289396		
			56.3	-1711.283909	-1711.052062	-1711.117981	-1711.296758	-1711.130830	1.47
			-169.6	-1711.278853	-1711.046871	-1711.112773	-1711.291933	-1711.125853	4.59

			-60.6	-1711.280910	-1711.049024	-1711.114875	-1711.294679	-1711.128644	2.84
		RS	72.7	-1711.282122	-1711.050152	-1711.115807	-1711.295708	-1711.129393	2.37
			171.9	-1711.275435	-1711.043471	-1711.109882	-1711.288301	-1711.122748	6.54
**4,4′**	4 of 5-yl semiquinone on 4 of semiquinone anion	*RR*	-49.2	-1711.281834	-1711.050240	-1711.115991	-1711.294677	-1711.128834	2.72
			-64.2	-1711.284323	-1711.052381	-1711.116594	-1711.297087	-1711.129358	2.39
			57.6	-1711.278596	-1711.046627	-1711.111390	-1711.289389	-1711.122183	6.90
			143.9	-1711.287106	-1711.055588	-1711.121506	-1711.298772	-1711.133172	0.00
		RS	-70.5	-1711.287495	-1711.055744	-1711.121043	-1711.298820	-1711.132368	0.50
			72.7	-1711.286288	-1711.054315	-1711.119464	-1711.298514	-1711.131690	0.93
			-177.2	-1711.277837	-1711.045951	-1711.110822	-1711.288398	-1711.121383	7.40
**4,7′**	4 of 5-yl semiquinone on 7 of semiquinone anion	*RR*	-56.1	-1711.283213	-1711.051346	-1711.116767	-1711.296016	-1711.129570	2.26
			61.7	-1711.273268	-1711.041387	-1711.107056	-1711.284061	-1711.117849	9.62
			158.5	-1711.283652	-1711.051768	-1711.116942	-1711.295122	-1711.128412	2.99
		RS	-83.2	-1711.283547	-1711.051895	-1711.117909	-1711.295393	-1711.129755	2.14
			82.5	-1711.278016	-1711.046337	-1711.112146	-1711.291090	-1711.125220	4.99
			59.4	-1711.280805	-1711.048829	-1711.112851	-1711.293873	-1711.125919	4.55
			166.6	-1711.251230	-1711.019857	-1711.085244	-1711.260984	-1711.094998	23.95
			-177.1	-1711.273617	-1711.041621	-1711.108334	-1711.284722	-1711.119439	8.62
	7 of 6-yl semiquinone on 4 of semiquinone anion	*RR*	-60.5	-1711.287773	-1711.055975	-1711.121137	-1711.299126	-1711.132490	0.43
			58.7	-1711.275350	-1711.043516	-1711.108853	-1711.286219	-1711.119722	8.44
			160.6	-1711.284984	saddle point		-1711.296549		
			154.6	-1711.284940	-1711.053254	-1711.118872	-1711.296235	-1711.130167	1.89
		RS	-83.1	-1711.286210	-1711.054664	-1711.120602	-1711.297300	-1711.131692	0.93
			80.2	-1711.280895	-1711.049241	-1711.114708	-1711.293067	-1711.126880	3.95
			58.9	-1711.283318	-1711.051356	-1711.115578	-1711.295624	-1711.127884	3.32
			179.6	-1711.255408	-1711.024083	-1711.089573	-1711.264536	-1711.098701	21.63
			-177.5	-1711.276590	-1711.044639	-1711.110102	-1711.286812	-1711.120324	8.06
**7,7′**	7 of 6-yl semiquinone on 7 of semiquinone anion	*RR*	-51.1	-1711.282639	-1711.050889	-1711.116412	-1711.294154	-1711.127927	3.29
			48.9	-1711.272179	-1711.040226	-1711.105395	-1711.283029	-1711.116245	10.62
			176.9	-1711.279815	-1711.047755	-1711.111779	-1711.292346	-1711.124310	5.56
			148.2	-1711.276890	-1711.045202	-1711.110886	-1711.289207	-1711.123203	6.26
		RS	-71.1	-1711.281993	-1711.050104	-1711.115635	-1711.294073	-1711.127715	3.42
			69.5	-1711.283970	-1711.052031	-1711.116918	-1711.295385	-1711.128333	3.04
			-175.0	-1711.270833	-1711.038971	-1711.104556	-1711.281760	-1711.115483	11.10

[a] Fiducial groups are the highest priority atoms on either side of the interring C-C bond.

[b] Electronic energy in water (including electrostatic PCM contributions).

[c] Sum of electronic and thermal enthalpies in water (including electrostatic PCM contributions).

[d] Sum of electronic and thermal free energies in water (including electrostatic PCM contributions).

[e] Electronic energy in water (including both electrostatic and non-electrostatic SMD contributions).

[f] $G_{SMD,RRHO} = G_{PCM,RRHO} – G_{el,PCM} + G_{el,SMD}$.

[g] Relative $G_{SMD,RRHO}$ values referred to the most stable structure identified in the series.

Thiomelanin synthesis and characterization. DHBT (50 mg, 0.3 mmol) was dissolved in MeOH (1 mL), then the proper buffer was added (0.05 M carbonate buffer pH 9, 30 mL). A solution of $K_3[Fe(CN)_6]$ (3 molar equivalent, 99 mg) in water was added. Suddenly, the reaction mixture turned deep red, then purple while a light grey solid separated from the aqueous media. The solid was recovered by centrifugation (7000 rpm, 4° C, 15 min), extensively washed with water (3 x 5 mL) and MeOH (2 x 3 mL), and then lyophilized. Thiomelanin was recovered as a greyish amorphous solid (yield: 35 mg, 70% w/w).

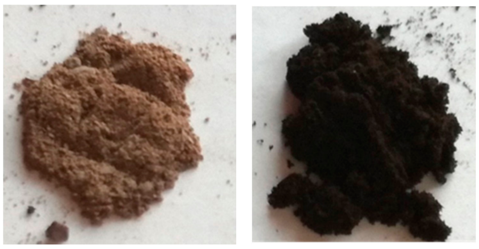

Figure S19. Powdered melanins collected by centrifugation of oxidation mixtures of DHBT (left) and DHI (right)

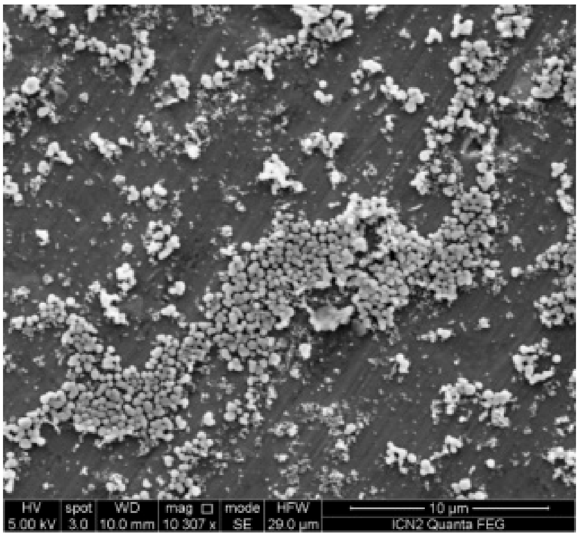

Figure S20. SEM image of thiomelanine powder.

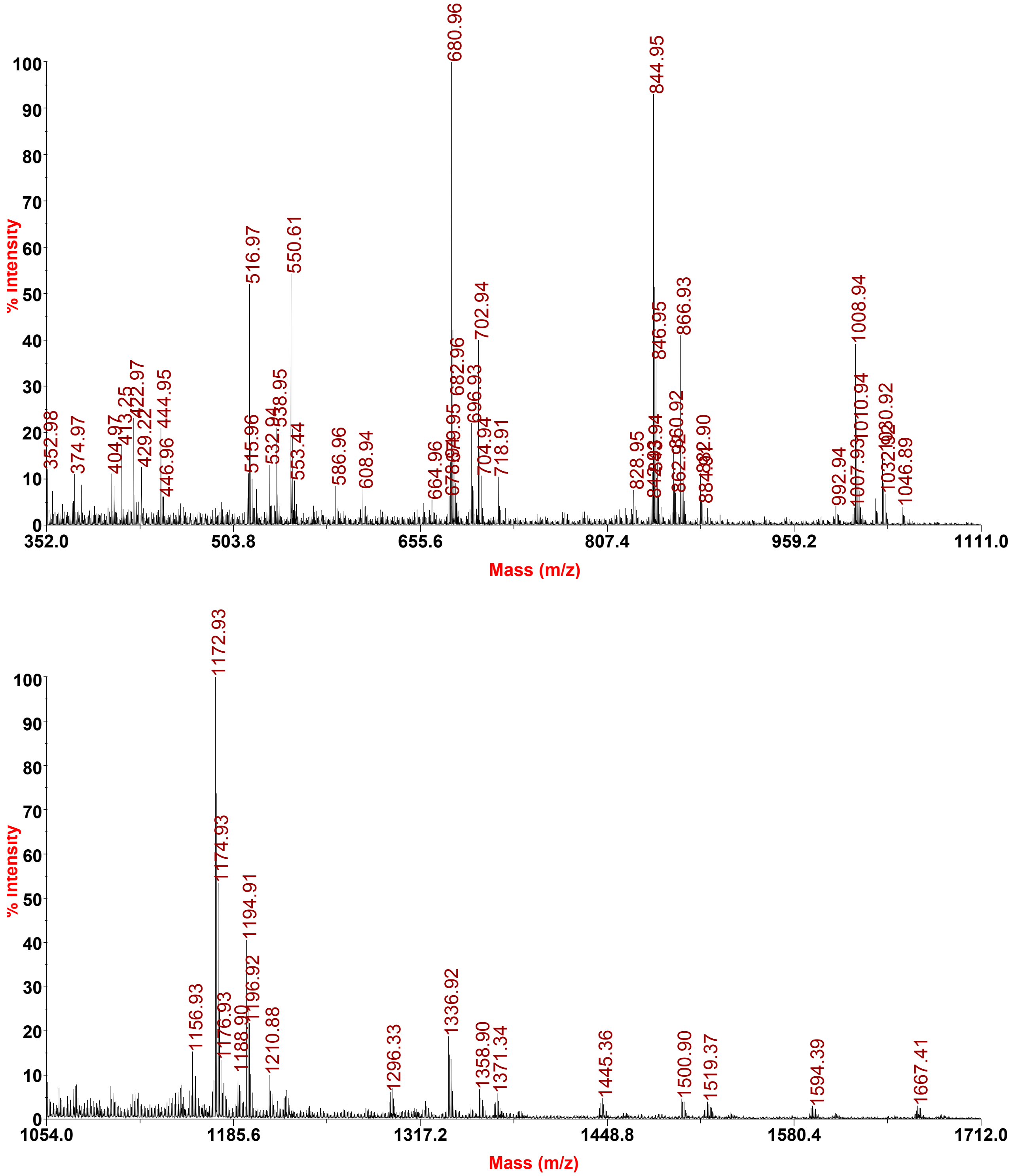

Figure S21. MALDI-MS spectrum of thiomelanin, showing 352 - 1111 *m*/*z* (top) and 1054 - 1712 *m*/*z* (bottom) spectral regions

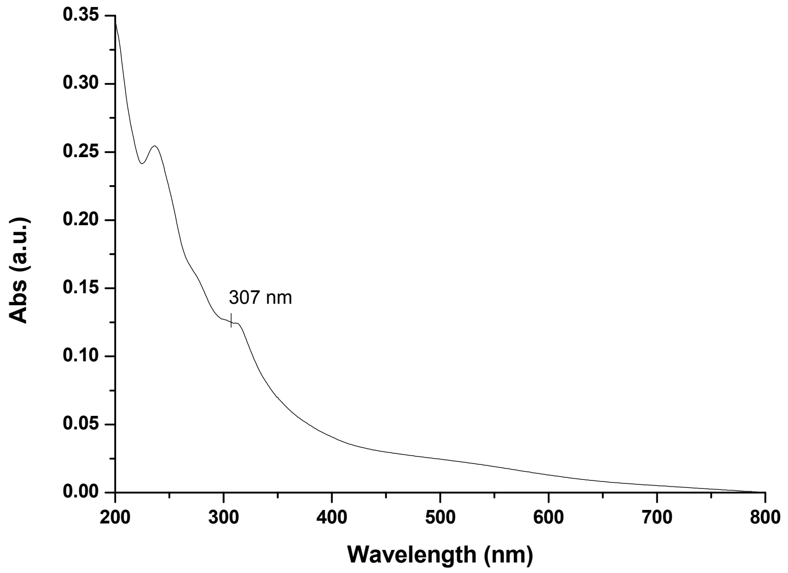

Figure S22. UV-Vis spectrum of thiomelanin as aqueous suspension (0.5 mg/mL).

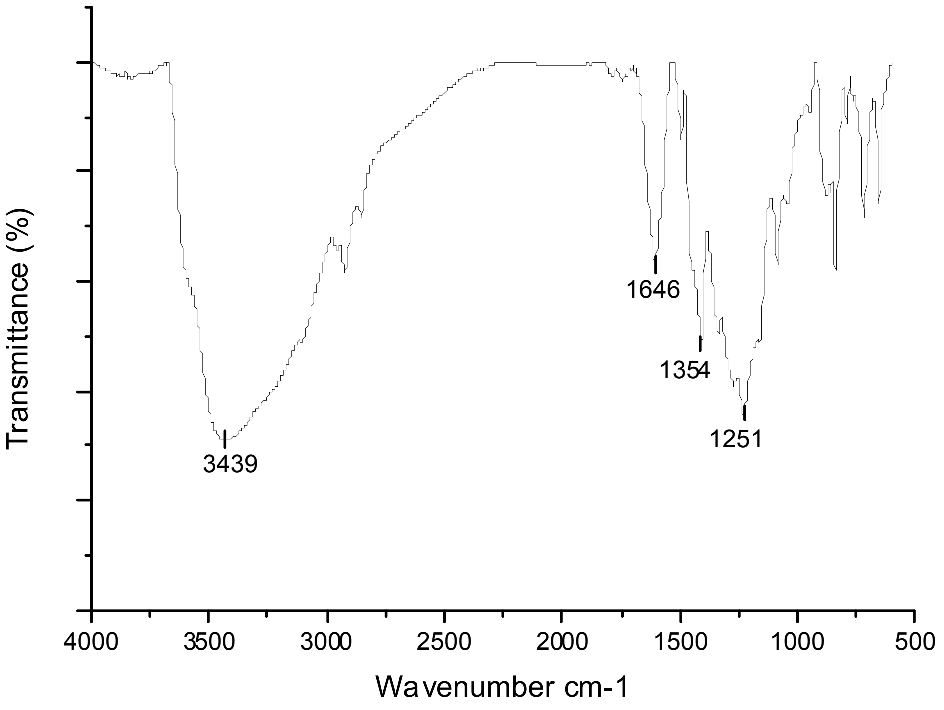

Figure S23. ATR/FT-IR spectrum of thiomelanin powder.

Coating experiments. DHBT or DHI were dissolved in methanol (3 mg/mL) and deposited on a clean substrate (glass or quartz) applying a standard spin-coating protocol (3000 rpm, 1 min, acc. 500 rpm/sec) and then exposed to gaseous ammonia vapors in a saturated chamber (15 min for DHI and 6 h for DHBT).
